# OncoMimic peptide-based immunotherapy EO2401 induces shared T cell clusters in patients with adrenal cancer

**DOI:** 10.1038/s43856-026-01755-8

**Published:** 2026-07-06

**Authors:** Christoph Schultheiß, Brenda Besemer, Edith Willscher, Lisa Paschold, Tifanny Mersceman, Alice Talpin, Clara Serger, Alfred Zippelius, Alfredo Berruti, Salvatore Grisanti, Catharina Willemien Menke-van der Houven van Oordt, Eric Baudin, Laura-Sophie Landwehr, Jaume Capdevila, Vivek Subbiah, Dan Granberg, Kirsten Gedske Daugaard, Alexandra Triebig, Thibault Gauduchon, Christine Do Cao, Marie-Eve Garcia, Joao Magalhaes, Laurent Chêne, Mascha Binder

**Affiliations:** 1 Division of Medical Oncology, University Hospital Basel, Basel, Switzerland; 2 Laboratory of Translational Immuno-Oncology, Department of Biomedicine, University and University Hospital Basel, Basel, Switzerland; 3 Department of Internal Medicine IV, Oncology/Hematology, Martin-Luther-University Halle-Wittenberg, Halle (Saale), Germany; 4 Enterome SA, Paris, France; 5 Laboratory of Cancer Immunology, Department of Biomedicine, University Hospital and University of Basel, Basel, Switzerland; 6 Università degli Studi di Brescia, Azienda Ospedaliera Spedali Civili, Brescia, Italy; 7 Department of Medical Oncology, Amsterdam UMC, Cancer Centre Amsterdam, Amsterdam, The Netherlands; 8 Endocrine Oncology, Gustave Roussy, Villejuif, France; 9 Internal Medicine, University Hospital Würzburg, Würzburg, Germany; 10 Vall d’Hebron University Hospital and Vall d’Hebron Institute of Oncology (VHIO), Barcelona, Spain; 11 Stanford Cancer Institute, Stanford University School of Medicine, Stanford, CA USA; 12 Department of Molecular Medicine and Surgery, Karolinska Institute, Stockholm, Karolinska University Hospital Solna, Stockholm, Sweden; 13 Medical Oncology, Rigshospitalet/University Hospital, Copenhagen, Denmark; 14 Medicine IV Endocrine Oncology, Ludwig Maximilians University of Munich, München, Germany; 15 Medical Oncology, Centre Léon Bérard, Lyon, France; 16 University Hospital Lille - Hôpital Claude Huriez, Lille, France; 17 Hospital Nord, Marseille, France; 18 Collaborative Research Institute Intelligent Oncology (CRIION), Freiburg, Germany

**Keywords:** Tumour immunology, Endocrine cancer, Tumour vaccines

## Abstract

**Background:**

Patients with advanced malignant adrenal tumors face poor prognoses with limited treatment options. Emerging data suggest that these rare tumors exhibit immunogenicity, potentially benefiting from intensified immunotherapy.

**Methods:**

The SPENCER trial (NCT04187404) evaluates EO2401, an off-the-shelf in vivo peptide immunotherapy containing three synthetic HLA-A*02 restricted-peptides called OncoMimic and derived from human gut commensals that exhibit molecular mimicry to tumor-associated antigens (BIRC5, FOXM1, IL13RA2) combined with nivolumab in patients with locally advanced or metastatic adrenocortical carcinoma (ACC) or malignant pheochromocytoma/ paraganglioma (PPGL). We performed T cell receptor (TCR) repertoire sequencing to profile the TCR architecture in ACC and characterize vaccination imprints.

**Results:**

Here we show that ACC patients are characterized by a restricted peripheral T cell richness. EO2401 does not expand highly shared TCR clones across patients but diversifies pre-existing T cell clusters. These clusters are not converging on known neoantigen-specific TCRs but exhibit generation probabilities characteristic of public clones some of which being shared across patients.

**Conclusions:**

These findings suggest that EO2401-based peptide immunotherapy combined with checkpoint inhibition can broaden and activate public, pre-existing T cell clusters, potentially enhancing tumor-specific immune responses.

## Plain language summary

Adrenal cancers are rare and often hard to treat. We wanted to understand whether a new cancer vaccine, called EO2401, can help the immune system recognize adrenal cancer. We studied blood samples from patients who received EO2401 together with nivolumab, a drug that can strengthen T cell responses against cancer. We found that patients with adrenal cancer had a reduced variety of T cells. After treatment, EO2401 appeared to diversify shared groups of T cells that may be able to recognize cancer-related targets. Our results suggest that EO2401 may help activate useful immune responses in adrenal cancer and should be explored further in future studies.

## Abstract (web summary)

Schultheiß et al profile longitudinal peripheral TCRβ repertoires in patients with adrenal cancer treated with oncomimetic peptide vaccine EO2401 and nivolumab. The data reveal adrenal cancer-associated repertoire restriction and EO2401-associated diversification of shared T cell clusters.

## Introduction

Adrenal cancers (ACs), including adrenocortical carcinoma (ACC) and malignant pheochromocytoma (PCC), are rare but aggressive malignancies with high mortality rates^1–3^. ACC arises from the adrenal cortex and may produce steroid hormones, leading to severe clinical manifestations such as Cushing’s syndrome and virilization^1,4^. Malignant PCC originates from the adrenal medulla, often associated with paroxysmal hypertension and catecholamine excess. Genetic predisposition is observed, including Li-Fraumeni syndrome, multiple endocrine neoplasia types 1 and 2, familial adenomatous polyposis, neurofibromatosis type 1, and SDHx mutation-related syndrome^5^.

Systemic treatment options for advanced adrenal tumors remain limited^1,6^. ACC is typically treated with mitotane, which suppresses steroidogenesis, alongside platin-based chemotherapy^2,7^. However, patients with metastatic disease have a five-year survival rate of only 10%. Malignant PCC exhibits a lower metastatic incidence (5–20%) but carries significant morbidity and long-term mortality, with treatment options such as multi-kinase inhibitors, alkylant-based chemotherapy, somatostatin analogs, and peptide receptor radionuclide therapy that remain palliative^8^.

Given these challenges, novel immunotherapeutic strategies are under active investigation. Therapeutic tumor vaccines aim to direct immune activation in combination with checkpoint blockade toward tumor-associated antigens (TAAs), enhancing tumor recognition and immune response^9–14^. Decades of research in autoimmunity and oncology have shown that certain microbial peptides closely resemble human peptides, and they can induce cross-reactive immune responses^15^.

OncoMimic peptides are highly prevalent microbiome-derived epitopes (found in >80% of individuals), carefully selected for their molecular similarity to TAAs, their strong immunogenicity and their ability to mobilize cross-reactive cytotoxic CD8 T cells, through an extensive pipeline combining in silico, in vitro, mouse in vivo and healthy human data^16^. Since these peptides are foreign epitopes, they retain immunogenicity even in patients who may be tolerant to the self (tumor) antigens. The high prevalence of OncoMimic peptides in the human gut microbiota implies frequent exposure of endogenous T cells throughout life. Such recurrent exposure is known to shape CD8 T cell repertoires including cytotoxic effector and memory clones and is likely to contribute to their differentiation into cytotoxic effector and memory subsets within the gut mucosa. In the context of cancer, such microbial antigens that mimic TAAs can be leveraged to trigger pre-existing memory T cell pools for rapid expansion and durable anti-tumor responses^15,17,18^. EO2401 is an investigational OncoMimic-based immunotherapy containing peptide mimics for cancer-associated antigens (BIRC5, FOXM1, IL13RA2). Each EO2401 mimic peptide has been shown to induce cross-reactive, IFN-γ-producing and cytotoxic CD8^+^ T cells recognizing the corresponding TAA in vitro, in HLA-A*02 transgenic mice, healthy individuals, and cancer patients^16^. The SPENCER trial (NCT04187404) investigates whether EO2401 enhances tumor immunogenicity and therapeutic synergy when combined with immune checkpoint inhibition in ACs. These questions are especially important in adrenal cancers, which are frequently characterized by immune-cold or immunosuppressive features, limited clinical benefit from checkpoint inhibition, and weakly defined biomarkers of response. Whether EO2401 can broaden or amplify tumor-relevant T cell immunity in this setting, either through direct expansion or diversification of tumor-reactive T cells or through secondary epitope spreading against additional tumor antigens, remains unknown.

Here, we profile the peripheral T cell receptor beta (TCRβ) repertoire architecture in adrenal cancer patients and describe immunogenetic repertoire imprints following EO2401 administration combined with nivolumab treatment in HLA-A*02-positive patients with advanced adrenal tumors. Our findings indicate the expansion of shared T cell clusters with public clonotype characteristics, supporting the potential of EO2401 immunotherapy to drive tumor-specific immune responses.

## Methods

### Study design and sample collection

The SPENCER trial (NCT04187404; registered at December 3, 2019 at ClinicalTrials.gov) is a multicenter study evaluating the safety and immunogenicity of EO2401 in combination with nivolumab in patients with advanced adrenal tumors. The trial was conducted at 12 centers in the United States, Denmark, France, Germany, Italy, Netherlands, Spain, and Sweden under approval of the local ethics committees and in compliance with the Declaration of Helsinki, but was terminated for strategic reasons. Blood samples used for the research described in the study comes from sampling of patients participating in the clinical study EOADR1-19/SPENCER (EudraCT number 2019-003396-19) where all sampling follows Good Clinical Practice via a clinical protocol approved by the Ethic Committees at the study sites. Specifically for the samples in the presented research the Ethic Committees approving the research include: CPP Ouest II Angers, Maison de la Recherche Clinique, CHU Angers 4, rue Larrey, 49933 Angers Cedex 9, France, no. identification RCB: 2019-003396-19, no. identification SI/CPP: 19.10.18.72055 (2020/25); CEIm-Comité de Ética de Investigación con medicamentos-Hospital Universitari Vall d’Hebron, Vall d’Hebron Institut de Recerca, Pg. Vall d’Hebron, 119-129, 08035 Barcelona, Spain, Comité decidió emitir Aprobado en la reunión celebrada el día 20/03/2020 (acta no. 418); Comitato Etico di Brescia, ASST degli Spedali Civili Azienda Ospedaliera Spedali Civili Piazzale Spedali Civili 1, 20123 Brescia, Italy, Numero del registro dei pareri del CE: 3891; Centrale Commissie Mensgebonden Onderzoek (CCMO), Parnassusplein 5, 2511 VX Den Haag, Netherlands, Ons kenmerk: CCMO20.0217/JvG/rm/71766, Betreft: Primair positief besluit NL71766.000.19; Etikprövningsmyndigheten, Box 2110, 750 02 Uppsala, Sweden, Dnr Ö 34-2020/3.1, Diarienummer 2019-06584. All participants were 18 years or older and provided written informed consent. The study protocol was approved by the local ethics committees and authorized by the competent authority. This translational study includes 11 HLA-A*02-positive patients treated in this trial. Peripheral blood mononuclear cells (PBMCs) were isolated before treatment initiation and at follow-up time points (6–14 weeks post-treatment). The 24 age-matched healthy donors and samples from the FOCUS trial assessing an anti-hTERT vaccine in head and neck cancer^19^ were included as controls.

### Ex vivo peptide stimulation and T cell expansion

PBMCs were thawed in thawing medium (IMDM supplemented with 2.5% heat inactivated human serum (hi HS), 1% Pennicillin-Streptomycin, 50 µM b-mercaptoethanol (b-ME) and 3 µg/mL of DNase I), washed (1300 rpm, 8 min, RT), counted and resuspended in T cell medium (TCM, IMDM supplemented with 10% hi HS, 1% PenStrep and 50 µM b-ME and seeded in either 24 well or 48 well plates at a respective density of 3.5–5 million cells or 2–3.4 million cells per well (respectively 500 µL or 250 µL per well), and cultured for 24 h (37 °C, 7.5% CO_2_). On day 1, peptides (HLA class I EO2316, EO2317 and EO2318) were added to the culture medium (final concentration of 1 µg/mL for each HLA class I peptide and 5 µg/mL for UCP2). On day 3, IL-2 was added to the culture medium (final concentration of 2 ng/mL, IL-2 reference: R&D 202-IL-010). On day 5, cells were split 1:3 and 2 ng/mL of IL-2 was added in a final volume of 2000 µL or 1000 µL for 24 or 48 well plates, respectively. On days 7 and 9, fresh TCM containing IL-2 (2 ng/mL) was added. If necessary, cells were split 1:2 at day nine. On day 12, cells were collected, and their viability and numbers were checked with an automated cell counter (Nucleo Counter NC-250) using an AO-DAPI staining reagent (solution 18 from Chemometec) before dry pellet preparation.

### Next-generation sequencing of T cell repertoires

Genomic (g)DNA was isolated from cryopreserved PBMCs or frozen pellets of in vitro expanded PBMCs or tetramer-sorted T cells using the GenElute Mammalian Genomic DNA Miniprep Kit (Sigma-Aldrich, Taufkirchen, Germany). To analyze immunogenetic imprints of EO2401 immunotherapy in AC patients and profile for vaccination-associated clonotypes, we performed V(D)J immunosequencing. For this, the rearranged T cell receptor beta chain (TRB) loci were amplified using 250 ng gDNA as template in a multiplex PCR as described^20–22^. After quality control on a 2100 Bioanalyzer (Agilent Technologies, Santa Clara, CA), sequencing and de-multiplexing was performed on the Illumina MiSeq platform (Illumina, San Diego, CA) with paired-end reads (2 ×301 cycles) at an average coverage of 62,000 reads per sample. The rearranged TRB loci were aligned to the reference genome using MiXCR (v3.0.12)^23^ and its default reference library. Only productive sequences with at least two read counts were considered for downstream analyses. A clone was identified as a unique CDR3 nucleotide sequence. For calculation of metrics and the V/VJ architecture as well as GLIPH2 clustering, repertoires were proportionally normalized to 50,000 read counts without discarding clonotypes.

### Immune repertoire analysis

We assessed TCRβ clonal repertoire architecture using three non-redundant and complementary metrics: TCR richness, clonality, and diversity. TCR richness refers to the total count of unique TCR clonotypes. Clonality serves as a measure of potential tumor-specific clonotype expansion or a proxy for an oligoclonal repertoire with limited diversity. It is quantified as 1 minus Pielou’s evenness index^24^, which gauges clonotype dominance in the repertoire (a value of 0 indicates maximum diversity, while a value of 1 indicates dominance by a single clonotype). To measure TCR diversity, we calculated Shannon entropy, which accounts for both the number of unique clonotypes (richness) and their relative frequencies (evenness). Shannon entropy was calculated using the formula ${{\rm{H}}}1=-{\sum }_{i=1}^{N}{{\rm{p}}}{\mbox{i}}\log ({{\rm{p}}}{\mbox{i}})$, where p_i_ represents the proportional frequency of each clonotype. Higher Shannon entropy values reflect a more diverse and evenly distributed repertoire, whereas lower values suggest clonal dominance or limited diversity. All analyses and data plotting were carried out using R (v4.3.3, R Core Team, 2024) and the packages tcR (v2.3.2) and immunarch (v0.9.1) as well as GraphPad Prism (version 10).

### TCR cluster identification and characterization

To map TCR clusters, we used the GLIPH2 algorithm, which identifies TCR groups with a high likelihood of shared antigen specificity^25,26^. It clusters TCR clonotypes based on the similarity of their CDR3 sequences, either by local motifs (2–4 amino acid sequences within the high-probability contact region of CDR3) or by global similarity (2–4 amino acid differences between two sequences). For input, we used all clonotypes from the respective analysis group. GLIPH2 was run using the following parameters: reference version 1.0, reference subset CD4+ and CD8+, and all_aa_interchangeable set to “yes”. The generation probability for each VDJ-rearranged clonotype was computed using the OLGA algorithm as approximation for the discrimination between public and private clonotypes^27^. OLGA pGen computation used olga v1.3.0 with the default human_T_beta generative model. Cluster motifs were cross-referenced against the human TRB content of VDJdb (release 2026-05-16; 88,003 human TRB entries)^28^. For each shared cluster motif, matching VDJdb/IEDB/neoTCR TRBβ CDR3 sequences were tabulated and classified by the antigen species of the cognate epitope into viral, human self/tumor-associated, or other specificities. Because the human self-antigen records of the current VDJdb release are heterogeneous and dominated by the cancer-testis antigen NY-ESO-1 as well as additionally containing autoimmune autoantigens, the TAA category was restricted to a curated set of bona fide HLA-A*02-restricted TAAs (NY-ESO-1, MLANA/MART1, PMEL, KLK3, IGF2BP2, BST2, TKT, SEC24A, PGT). For the antigen-attribution heat map, the VDJdb-annotated motifs were ranked by their FU/BL fold-change in cumulative cluster richness, and the most strongly diversifying motifs were displayed against the curated TAAs of their matching VDJdb TCRs. Because VDJdb-annotated motifs were on average shorter than unannotated motifs, the association between diversification and VDJdb annotation was additionally evaluated after regressing out motif length; this length-residual comparison was used to qualify the antigen-attribution map as hypothesis-generating rather than confirmatory. This matching approach was cross-referenced the IEDB database (187,488 entries, ~95% virus-related)^29^ and the neoTCR database^30^ covering 954 neoantigen-directed TCRs (data release 2022-12-20). All analyses were performed in Python (v3.10) using numpy (v1.26), pandas (v2.2), scipy (v1.15), statsmodels (v0.14), matplotlib (v3.8), matplotlib-venn (v1.1) and openpyxl (v3.1).

### Statistics and reproducibility

To compare immune repertoire metrics across multiple groups, we used one-way ANOVA followed by Tukey’s multiple comparisons test. Paired comparisons were performed using the non-parametric Wilcoxon matched-pairs signed rank test. Overlap between in-hominis and ex vivo GLIPH2 motif catalogues was assessed using a two-set comparison of motifs identified in the post-vaccination patient repertoire and after EO2401 peptide-pool stimulation. Statistical significance was evaluated by a one-sided hypergeometric test, $P\left(X\ge x\right)={\sum }_{k=x}^{\min \left(a,b\right)}\frac{\left(\frac{a}{k}\right)\left(\frac{N-a}{b-k}\right)}{\left(\frac{N}{b}\right)}$, where N denotes the assumed motif universe size, a and b the numbers of motifs in the two catalogues, and x the observed overlap. Because the true GLIPH2 motif universe is not fixed, analyses were repeated across plausible universe sizes of 500–5000 motifs. To test for cluster bias towards high-probability clonotypes, we analyzed vaccination-expanded versus background clonotypes for paired SPENCER patients. Unique CDR3 amino acid sequences with paired V/J usage were tabulated, and a clonotype was considered expanded at FU if it was absent at BL and detectable at FU or showed a frequency increase Δfreq >1 × 10⁻⁴ in favor of FU. For each patient, a control set of equal size was randomly sampled from BL clonotypes that did not meet the expansion criterion. To limit runtime, both expanded and control sets were down-sampled to 500 clonotypes. Group distributions were compared using a two-sided Mann–Whitney U test, and effect size was estimated with Cliff’s δ. To assess EO2401 cluster selectivity, the cumulative per-sample frequency of cluster motifs was defined as the sum of cloneFraction over all unique CDR3aa containing at least one of the 32 shared motifs. A random control motif set was constructed by repeatedly drawing 200 unique substrings from baseline AC CDR3aa with lengths matched to the empirical distribution of the 32 shared motif lengths and with one position randomly replaced by the wildcard symbol “%”. The identical scanning procedure was applied to the random k-mer set. Cohort comparisons used the two-sided Mann–Whitney U test. TRBV gene usage of unique cluster-contributing CDR3 sequences was compared with the TRBV gene distribution of the full repertoire background of the corresponding SPENCER samples. For each TRBV family, enrichment among cluster members was tested with a one-sided Fisher’s exact test, and *p*-values were Benjamini-Hochberg-adjusted across all tested V genes. TRBV families represented by fewer than five cluster-member sequences were excluded from significance testing.

### Ethics approval

The SPENCER trial (NCT04187404) was registered at December 3, 2019 at ClinicalTrials.gov. This study was performed in accordance with the Declaration of Helsinki. All participants were 18 years or older and provided written informed consent. The study protocol was approved by the local ethics committees and authorized by the competent authority. Specifically for the samples in the presented research the Ethic Committees approving the research include: CPP Ouest II Angers, Maison de la Recherche Clinique, CHU Angers 4, rue Larrey, 49933 Angers Cedex 9, France, no. identification RCB: 2019-003396-19, no. identification SI/CPP: 19.10.18.72055 (2020/25); CEIm-Comité de Ética de Investigación con medicamentos-Hospital Universitari Vall d’Hebron, Vall d’Hebron Institut de Recerca, Pg. Vall d’Hebron, 119-129, 08035 Barcelona, Spain, Comité decidió emitir Aprobado en la reunión celebrada el día 20/03/2020 (acta no. 418); Comitato Etico di Brescia, ASST degli Spedali Civili Azienda Ospedaliera Spedali Civili Piazzale Spedali Civili 1, 20123 Brescia, Italy, Numero del registro dei pareri del CE: 3891; Centrale Commissie Mensgebonden Onderzoek (CCMO), Parnassusplein 5, 2511 VX Den Haag, Netherlands, Ons kenmerk: CCMO20.0217/JvG/rm/71766, Betreft: Primair positief besluit NL71766.000.19; Etikprövningsmyndigheten, Box 2110, 750 02 Uppsala, Sweden, Dnr Ö 34-2020/3.1, Diarienummer 2019-06584. Equivalent approvals and permissions to access and analyze deidentified patient records were obtained from the respective ethics committees at all participating sites (documentation available upon request). All patients provided written informed consent before enrollment and before undergoing any study-specific procedures [ClinicalTrials.gov identifier: NCT04187404].

## Results

### Baseline and on-treatment T cell immune metrics of patients treated on the SPENCER trial

**Fig. 1:**
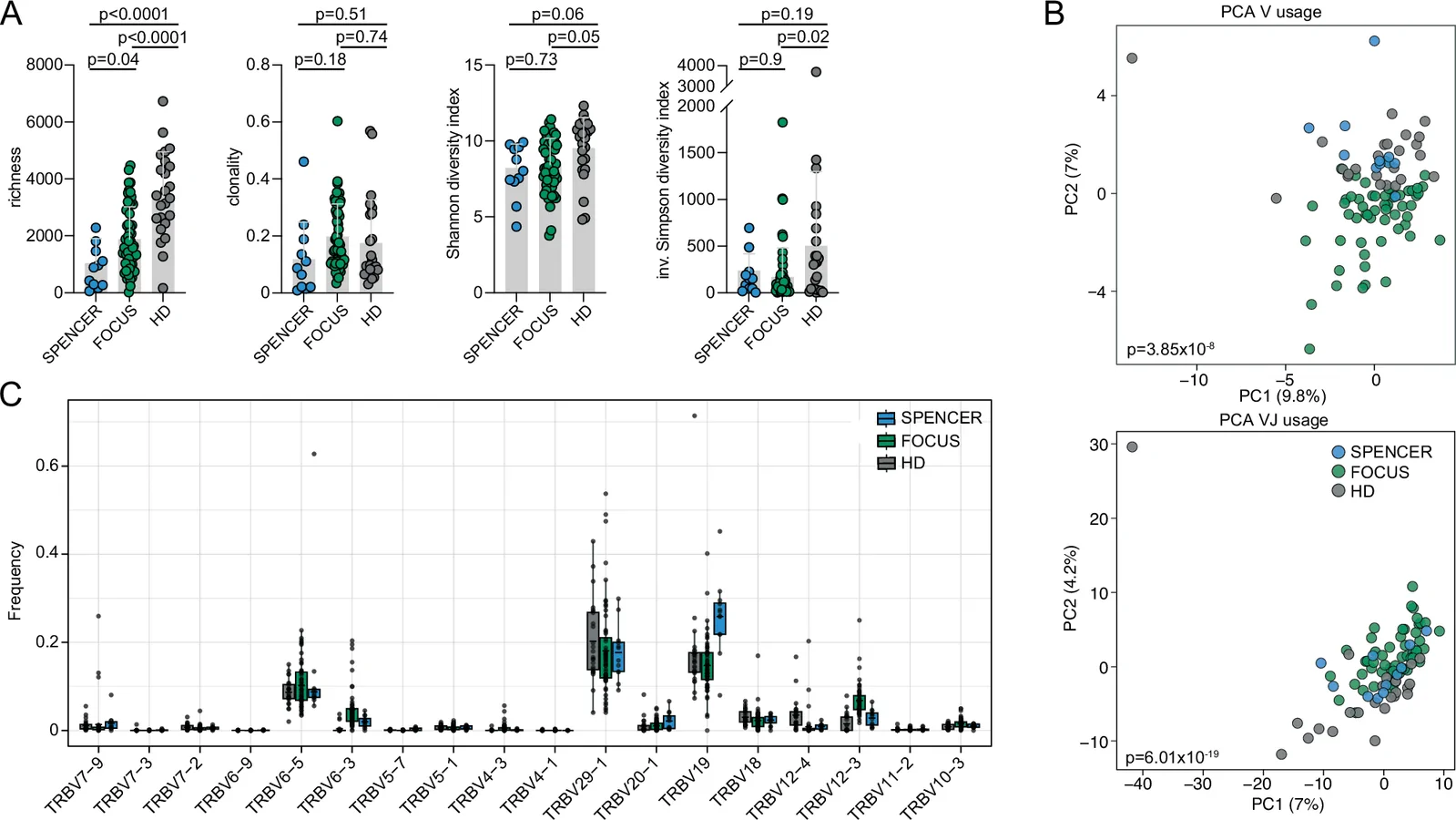
Immune repertoire metrics of peripheral T cells in the SPENCER cohort. **A** T cell repertoire richness, clonality, and diversity in patients with adrenal cancer from the SPENCER study at baseline (*n* = 11) as compared to the FOCUS (*n* = 56) and healthy (HD; *n* = 24) control cohorts. Gray bars represent the mean ± SD. Statistics: One-way ANOVA with Tukey’s post-hoc test. **B** PCA of TRBV and TRBV-J gene usage in the SPENCER, FOCUS, and HD cohorts. Statistical analysis: Pillai–Bartlett test of multivariate analysis of variance (MANOVA) of all principal components. **C** Mean frequency of TRBV gene usage for the selected V gene families. Each dot represents one individual sample. Boxes indicate the interquartile range, with box limits corresponding to the 25th and 75th percentiles. Whiskers extend to the most extreme values within 1.5× interquartile range.

Our baseline (BL) adrenal cancer (AC) cohort from the SPENCER trial encompassed 11 HLA-A*02-positive individuals (Table 1). To first investigate the peripheral T cell architecture in adrenal cancers, we profiled AC TCR metrics at BL alongside blood from healthy individuals (HD) and patients with head and neck squamous cell carcinoma (HNSCC) receiving anti-PD1 treatment from the FOCUS trial^19,31^ as a cancer control cohort. As expected for the immunosuppressive, frequently cortisol-secreting environment of AC, a subset of AC samples yielded lower raw read counts than the control cohorts (Supplementary Fig. 1, Supplementary Data 1). To exclude this as major confounder for the repertoire comparisons, we performed rarefaction analyses. This revealed that clonotype richness increased with sequencing depth, but AC repertoires remained clearly less rich than HD at comparable sample sizes, indicating a biological rather than sampling effect, while Shannon diversity tracked nearly identically across cohorts when plotted against accumulated clonotypes (Supplementary Fig. 1). T cell richness was markedly reduced in all cancer patients, but patients with AC exhibited the lowest levels (Fig. 1A). While clonality in AC tended to be lower than in HD and HNSCC, overall diversity measures were modestly but similarly reduced across both cancer cohorts compared to HD, suggesting a cancer-associated loss of rare clonotypes (Fig. 1A). Notably, the inverse Simpson calculation indicated higher clonal dominance in the FOCUS cohort, pointing to more pronounced expansion of select clones (Fig. 1A). Principal component analysis (PCA) of global V and VJ gene usage revealed no major repertoire skewing between AC and HD, although V gene usage showed a trend toward separation of AC and HNSCC with HD distributed across both (Fig. 1B). VJ usage, in contrast, indicated a subtle divergence of both cancer cohorts from HDs (Fig. 1B). Together, this suggested some cancer- or even cancer entity-related T cell receptor skewing in these cohorts. Cross-cohort comparisons of V gene frequencies identified selective enrichment of TRBV19 and increased TRBV20-1 in AC and elevated TRBV12-3 in HNSCC (Fig. 1C). In addition, TRBV6-3 usage was increased in both AC and HNSCC, while TRBV12-4 was reduced relative to HD (Fig. 1C).

**Table 1:** Cohort characteristics

	Healthy individuals	Adrenal cancer patients	HNSCC patients
Nb of patients	24	11	56
Nb of samples	24	42	56
Age median (range)	50.5 years (24–73)	46 years (30–73)	62 years (48–88)
Sex (female/male)	15/8		12/44

### Immunogenetic imprints of EO2401-mimic administration in AC patients

**Fig. 2:**
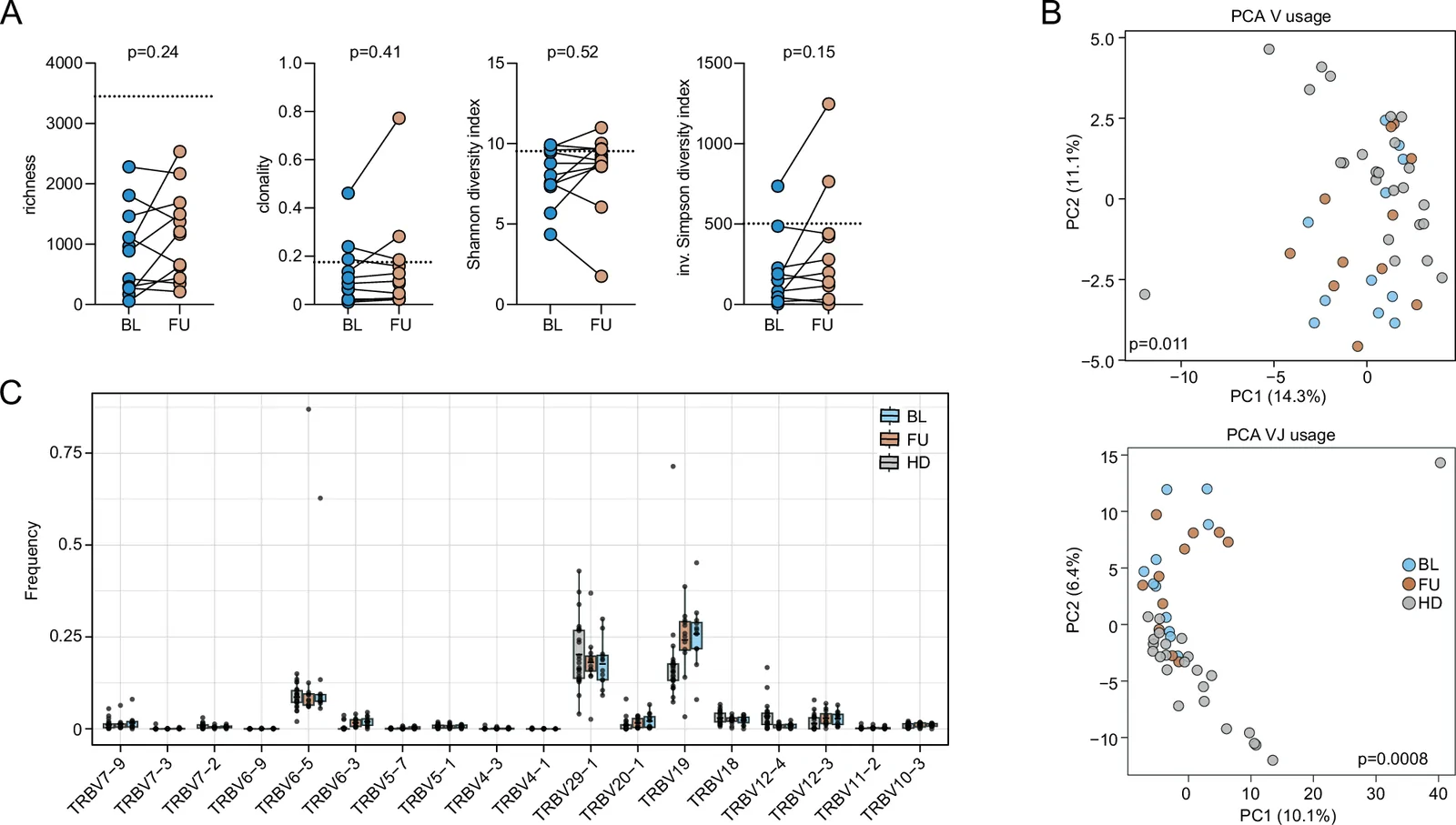
Post-treatment immune repertoire metrics in the SPENCER cohort. **A** T cell repertoire richness, clonality, and diversity measures in patients with adrenal cancer from the SPENCER study at baseline (BL;* n* = 11) and post-treatment (FU; *n* = 11). Only individuals with matched FU or in vitro expansion data (Fig. 4) were included. Dotted lines indicate mean value in healthy individuals. Statistics: Wilcoxon matched-pairs signed rank test. **B** PCA of TRBV and TRBV-J gene usage in the SPENCER cohort pre-/post-treatment as compared to HDs. Statistical analysis: Pillai-Bartlett test of multivariate analysis of variance (MANOVA) of all principal components. **C** Mean frequency of TRBV gene usage for the selected V gene families. Each dot represents one individual sample. Boxes indicate the interquartile range, with box limits corresponding to the 25th and 75th percentiles. Whiskers extend to the most extreme values within 1.5× interquartile range.

We next assessed global shifts in TCR repertoire metrics upon EO2401 administration. While individual patients showed increased richness (median: 888 at BL to 1185 clonotypes) and diversity (median Shannon: 8 at BL to 9; median inv. Simpson: 151 at BL to 240) post-treatment (Follow-up/FU), group-level metrics at FU generally remained below the mean values observed in HD (Fig. 2A). The absence of a broad reshaping of the peripheral immune architecture by the EO2401 treatment was also mirrored by the unaltered V and VJ gene usage analyses (Fig. 2B, C).

**Fig. 3:**
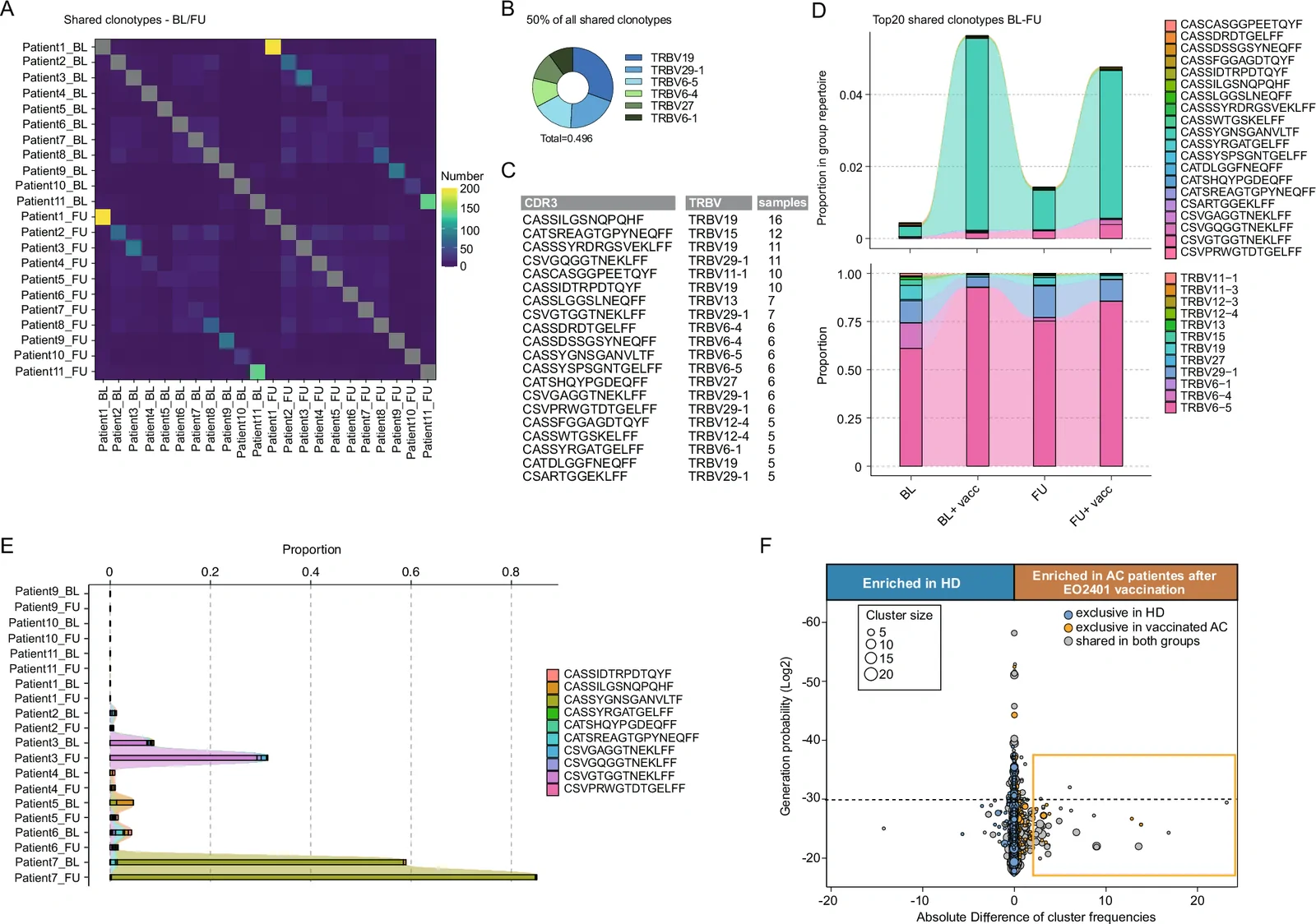
EO2401-induced immune repertoire imprints in patients with adrenal cancer. **A** Inter- and intra-patient clonal overlap of unique TCR CDR3 clonotypes at baseline (BL) and after EO2401 treatment (FU) displayed as a heat map. **B** Top 6 used TRBV genes within all shared TCR clonotypes. **C** Top20 most frequently shared TCR clonotypes between BL/FU samples. **D** Clonal tracking of top20 shared clonotypes at indicated time points, either for unique TCRs (upper panel) or for used TRBV genes (lower panel). The “+vacc” settings indicate in vitro peptide stimulation of the respective BL or FU sample. **E** Clonal tracking of the indicated 10 clonotypes with the highest frequencies at BL and FU. **F** Distribution and generation probability of GLIPH2-calculated TCR clusters in EO2401-treated adrenal cancer (AC) patients as compared to healthy controls. Dot sizes reflect the number of individuals contributing TCR clonotypes to a distinct cluster. Clusters exclusively found in EO2401-treated AC patients in orange, those exclusively found in HDs in blue, and those found in both groups are colored in gray.

To evaluate the response to EO2401-mimic immunotherapy in more detail, we next identified shared clonotypes between the 11 patients with complete BL and FU samples. Shared clonotypes were defined as identical productive CDR3 amino acid sequences detected in samples from ≥2 individuals (inter-patient sharing) or ≥2 time points of the same individual (intra-patient sharing). We found a total of 1,070 unique shared clonotypes, but despite the identical HLA-A*02 background no consistent enrichment of individual clonotypes was observed at FU (Fig. 3A). Clonotypes were mainly shared on the individual level, although some general level of sharedness within the BL and FU cohorts was observed indicating an overlapping public TCR space in the HLA-A*02 background. Six TRBV gene families accounted for half of all shared clonotypes with TRBV19 (173 clonotypes), TRBV29-1 (115 clonotypes), and TRBV6-5 (90 clonotypes) configurations being the most frequently represented (Fig. 3B). The 20 most commonly shared clonotypes mirrored this pattern (Fig. 3C) but lacked consistent clonal expansion across patients (Fig. 3D, E). Notably, we also mined the VDJdb^28^, neoTCR^30^ and IEDB^29^ databases for exact matches of TCRs with known neoantigen reactivity, but did not detect shared enrichment of such TCRs.

Given the lack of dominant shared clonal expansion patterns after treatment with EO2401, we used GLIPH2 to identify TCR clusters with predicted shared antigen specificity in post-treatment repertoires (Fig. 3F). GLIPH2 is a computational tool that groups TCR sequences in clusters based on shared CDR3 paratope motifs and predicted HLA-restricted antigen recognition, allowing inference of convergent immune responses^25,26^. Cluster frequencies were calculated as the sum of clonal frequencies within each cluster, and CDR3 generation probabilities were computed using the OLGA algorithm^27^ to distinguish public from private clusters. This analysis revealed 61 shared and expanded TCR clusters in AC patients following treatment (Fig. 3F, Supplementary Table 1). The largest clusters were composed of clonotypes contributed by up to five individuals. Notably, most clusters had relatively high generation probabilities consistent with a public character of the cluster-defining CDR3 motifs. In contrast, healthy controls showed no comparable clustering despite their greater repertoire richness supporting an EO2401-associated origin of the observed TCR motifs.

### In vitro expansion of EO2401-reactive T cells

**Fig. 4:**
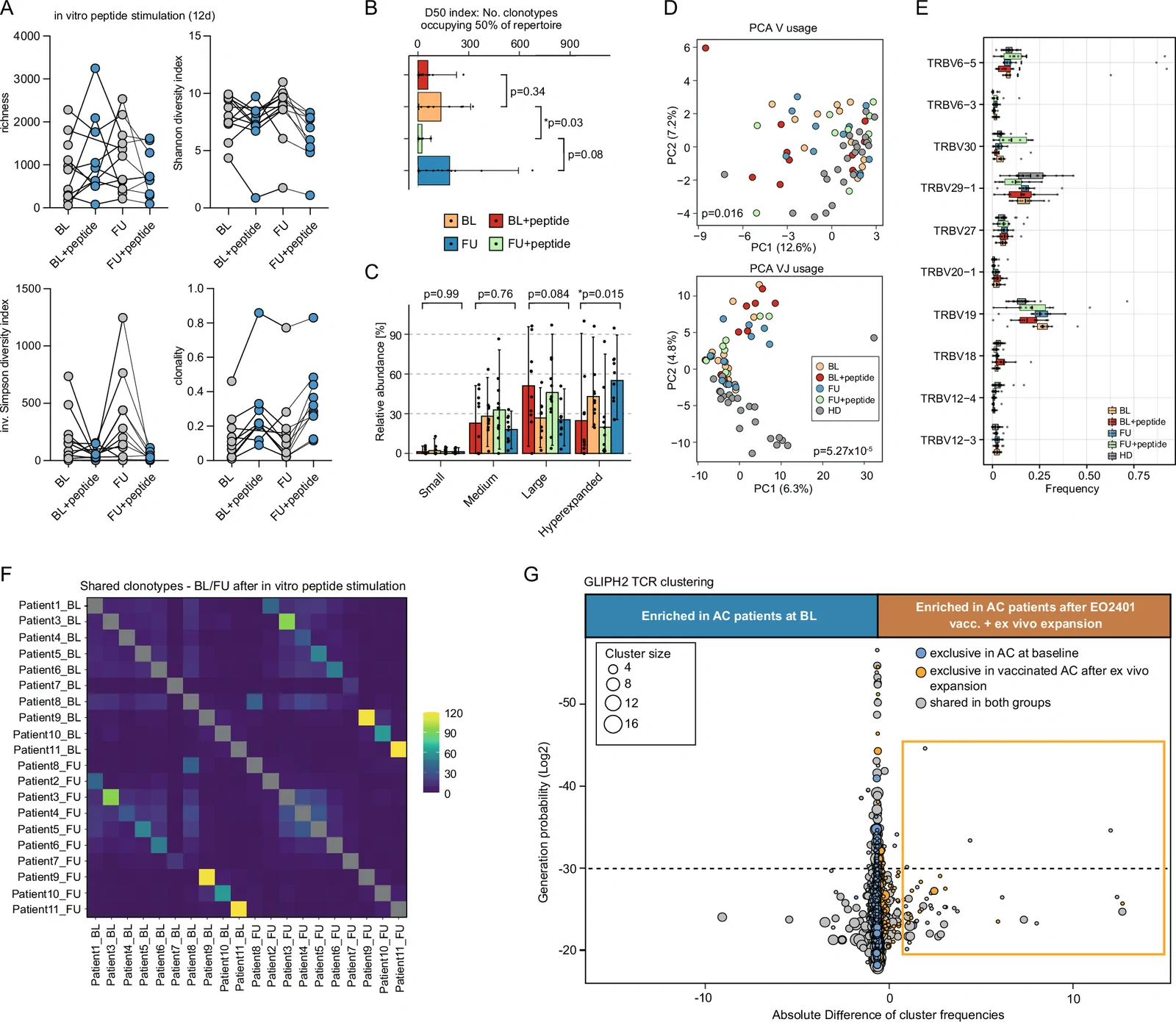
In vitro expansion of EO2401-reactive TCR clonotypes. **A** T cell repertoire richness, clonality, and diversity measures in patients with adrenal cancer from the SPENCER study at baseline (BL;* n* = 11) and post-treatment (FU; *n* = 12) and after in vitro stimulation with EO2401 peptide pool. **B** Mean ( ± SD) number of TCR clonotypes accounting for 50% of the repertoire space (D50 index). **C** Mean (±SD) summary proportion of clonotypes according to their size within the repertoire. Clone size definition: Hyperexpanded = 0.01 < x; large = 0.001 < x ≤ 0.01; Medium = 1 × 10^−4^ < x ≤ 0.001; Small = 0 < x ≤ 1 × 10^−4^. **D** PCA of TRBV and TRBV-J gene usage in the indicated pre-/post-treatment or ex vivo stimulation settings as compared to HDs. Statistical analysis: Pillai-Bartlett test of multivariate analysis of variance (MANOVA) of all principal components. **E** Mean frequency of TRBV gene usage for the selected V gene families. Each dot represents one individual sample. Boxes indicate the interquartile range, with box limits corresponding to the 25th and 75th percentiles. Whiskers extend to the most extreme values within 1.5× interquartile range. **F** Inter- and intra-patient clonal overlap of unique TCR CDR3 clonotypes at in vitro stimulated baseline (BL) and post-EO2401 treatment (FU) samples displayed as a heat map. **G** Distribution and generation probability of GLIPH2-calculated TCR clusters in ex vivo peptide-stimulated samples from EO2401-treated adrenal cancer (AC) patients as compared to AC baseline (BL) samples. Dot sizes reflect the number of individuals contributing TCR clonotypes to a distinct cluster. Clusters exclusively found in ex vivo stimulated samples in orange, those exclusively found in BL samples in blue, and those found in both groups are colored in gray.

To validate EO2401-specificity of TCR clusters, we next stimulated PBMCs derived from 10 AC patients at BL or FU with the EO2401 peptide pool in vitro. As shown in Fig. 4, in vitro stimulation had no clear impact on repertoire richness but displayed trends towards lower diversity measures and increased clonality independent of pre-treatment status (Fig. 4A). This was accompanied by changes in clonal abundancy measures indicating some degree of clonal expansion in the ex vivo stimulated samples (Fig. 4B, C). However, PCA analysis of the global TRBV and TRBV-J architecture as well as frequencies of individual TRBV genes did not reveal induction of an EO2401-specific pattern (Fig. 4D, E). Consistent with our findings in the unexpanded repertoires, profiling for shared clonotypes after stimulation again yielded a dominant intra-patient clonal sharing pattern (Fig. 4F). GLIPH2 clustering identified 51 enriched TCR clusters with relatively high generation probability after in vitro stimulation (Fig. 4G, Supplementary Table 2).

### Selectivity and features of EO2401-associated T cell clusters

**Fig. 5:**
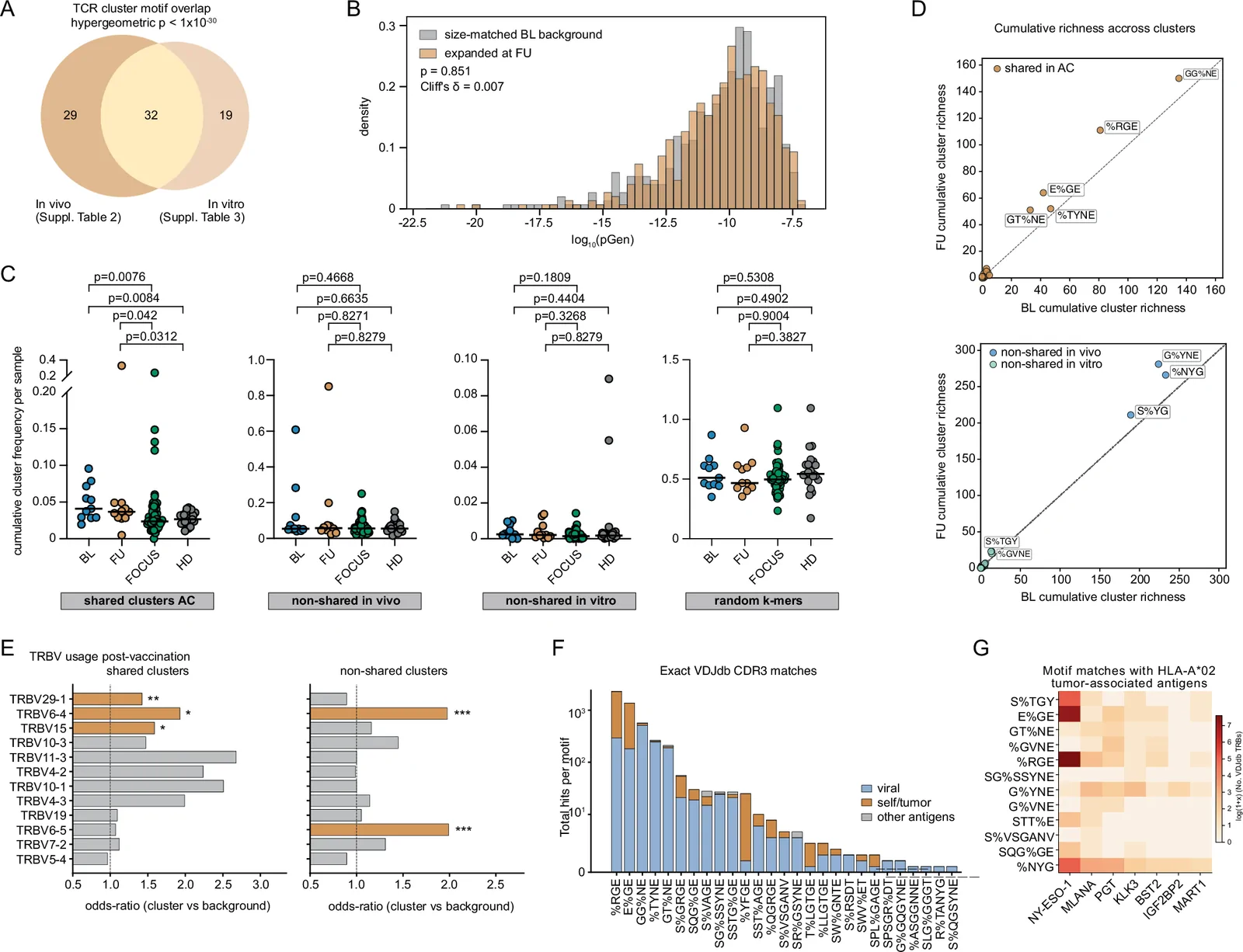
Selectivity and sequence features of EO2401-associated T cell clusters. **A** Overlap of the GLIPH2 cluster motifs identified in the post-vaccination repertoires (Supplementary Table 1, 61 motifs) and after in vitro EO2401 peptide stimulation (Supplementary Table 2, 51 motifs). **B** Distribution of OLGA-derived generation probability (log_10_(pGen)) for clonotypes expanded between BL and FU across the 11 paired SPENCER patients (orange, *n* = 495) and a per-patient size-matched random baseline-background control set (gray, *n* = 495). Statistics: two-sided Mann–Whitney U test and Cliff’s δ. **C** Median cumulative per-sample frequency of CDR3 sequences matching the 32 shared cluster motifs, the non-shared in vivo-only motifs, the non-shared in vitro-only motifs, or 200 random length-matched control k-mer motifs, shown for SPENCER BL (*n* = 11), FU (*n* = 11), FOCUS (*n* = 56) and HDs (*n* = 23). Statistics: two-sided Mann–Whitney U test. **D** Cumulative cluster-member richness (sum of unique CDR3 sequences across the 11 paired SPENCER patients) at BL versus FU for the shared clusters (top) and for the non-shared in vivo/in vitro-only clusters (bottom). Points above the dashed line correspond to clusters that recruited additional CDR3 clonotypes after vaccination. **E** TRBV-family enrichment of unique CDR3 cluster sequences against the full repertoire background, shown separately for the shared clusters (left) and the non-shared clusters (right). One-sided Fisher’s exact test, Benjamini-Hochberg-adjusted across V genes; *p*_adj_ < 0.05 (*), *p*_adj_ <0.01 (**), *p*_adj_ <0.001 (***). **F** Number of VDJdb CDR3 matches per shared cluster motif, stratified by antigen category into viral, TAA, and other. **G** Heat map of the most strongly diversifying VDJdb-annotated cluster motifs (rows, ranked by FU/BL fold-change in cumulative cluster richness) against the curated HLA-A*02-restricted TAAs of their matching VDJdb TCRs (columns). Cell color: log(1 + x) of the number of matching VDJdb TRBs.

To address whether the GLIPH2 clusters identified after EO2401 administration represent a genuine vaccine-related imprint rather than stochastic sampling artifact of public TCR architectures, we characterized the post-treatment cluster catalogue in more detail (Fig. 5). Of the 51 clusters enriched after in vitro EO2401 peptide stimulation, 32 (63%) were also present in the independently derived post-vaccination catalogue which represents a 10- to 50-fold enrichment over chance (hypergeometric *p* < 1 × 10^−30^; Fig. 5A). Recovery of the same CDR3 motifs by two independent procedures is consistent with an OncoMimic-driven imprint rather than a bystander or sampling effect. Consistent with this, the distribution of OLGA-derived generation probabilities of clonotypes expanded after vaccination were indistinguishable from those of a size-matched baseline background set (median log_10_ pGen −10.00 vs −9.92; Mann–Whitney *p* = 0.85; Cliff’s δ = 0.007), suggesting no bias toward intrinsically high-probability public clonotypes (Fig. 5B). We next corroborated the AC selectivity of these motifs by quantifying, per sample, the cumulative repertoire frequency of CDR3 sequences matching the 32 shared cluster motifs. Shared cluster motifs were notedly more frequent in adrenal cancer than in healthy donor or FOCUS repertoires at both BL and FU, whereas neither the non-shared (in vivo/in vitro-only) cluster motifs nor 200 random length-matched control k-mers showed comparable enrichment (Fig. 5C). The cohort-level selectivity signal was therefore confined to the convergently recovered shared clusters.

Although the cumulative clonal frequency of the clusters did not increase from BL to FU, the clusters diversified: 13 of 32 shared clusters gained unique low-frequency CDR3 members after vaccination, most pronounced for the %RGE, E%GE, GT%NE, %TYNE and GG%NE motifs (Fig. 5D). A smaller proportion of non-shared clusters showed the same trend (9/29 in vivo, 5/19 in vitro) (Fig. 5D). This pattern indicates recruitment of additional clonotypes into pre-existing cluster architectures rather than amplification of dominant clones. TRBV-gene analysis of the diversifying clusters revealed a biased TRBV usage: shared clusters were enriched for TRBV29-1, TRBV6-4 and TRBV15, while non-shared clusters carried a strong TRBV6-family signature (TRBV6-5, TRBV6-3, TRBV6-4) (Fig. 5E).

Finally, we mapped the 32 shared cluster motifs against the human TRB content of VDJdb^28^. Twenty-seven motifs (84%) matched CDR3 sequences of known viral antigen-specific CD8 TCRs, and 19 (59%) matched TCRs specific for bona fide HLA-A*02-restricted TAAs (Fig. 5F; Supplementary Data 2). The most strongly diversifying motifs architecturally resembled HLA-A*02-restricted TCRs against TAAs, predominantly the cancer-testis antigen NY-ESO-1 and the melanoma antigens MLANA/MART1 and PMEL, as well as KLK3, BST2 and IGF2BP2 (Fig. 5G). The viral architectural overlap was independently reproduced when the shared cluster motifs were cross-referenced against the IEDB database^29^ (27/32 motifs matching viral antigen-specific CD8 TCRs in both databases; Supplementary Fig. 2A), whereas the TAA signal was database-dependent and scarce for IEDB due to the biased catalogue (Supplementary Data 3). In addition, cross-referencing the shared cluster motifs against neoantigen-specific TCRs in the neoTCR database^30^ returned only a sparse signal: 5 of the 32 motifs (exclusively the shortest, J-proximal motifs) matched 19 neoantigen-specific TCRs directed against 18 distinct neoantigens, with no convergence onto a shared neoantigen and no HLA-A*02 enrichment (Supplementary Fig. 2B, Supplementary Data 4). The total number of matches did not exceed that of random length-matched control motifs.

Together, the EO2401-associated cluster architecture shows no specific resemblance to catalogued neoantigen-reactive TCRs, consistent with EO2401 targeting shared HLA-A*02-restricted TAAs (BIRC5, FOXM1 and IL13RA2) rather than patient-private neoantigens.

## Discussion

Our findings demonstrate that administration of EO2401, a microbiome-derived peptide immunotherapy, can elicit the broadening of shared pre-existing peripheral T cell clusters in patients with adrenal cancer suggesting activation and diversification of public immune responses. These results support the central mechanistic premise of OncoMimic peptide immunotherapies: leveraging microbial mimicry to mobilize pre-existing memory T cells against TAAs, with the goal of engaging otherwise immune-cold tumors such as adrenal carcinomas^16^.

Previous studies have shown that commensal bacteria can shape systemic immunity and stimulate antitumor responses through T cell cross-reactivity between microbial and tumor-derived peptides^32^. These findings provide a rationale for leveraging molecular mimicry to enhance the immunogenicity of traditional cancer vaccines, potentially overcoming immune tolerance mechanisms against tumor-associated self-antigens. Consistent with this concept, interim results from the SPENCER trial demonstrated EO2401-specific T cell responses in 91% of patients, with the magnitude of the induced immune response correlating with tumor shrinkage^33^. Similar findings have been reported in glioblastoma, another highly immunosuppressive tumor type as AC, where EO2401-specific CD8^+^ T cells were detected after vaccination by ELISpot in all evaluable patients and ex vivo by tetramer staining in 19/20 patients^34,35^. Importantly, these T cells showed confirmed cross-reactivity against the corresponding TAAs in 89–95% of patients^34,35^. Moreover, related vaccination approaches using microbiome-derived peptides have likewise elicited robust CD8^+^ T cell responses^18^, further supporting the feasibility of molecular mimicry-based vaccination strategies in highly immunoevasive cancers. Collectively, these findings indicate that EO2401 can induce functional, cross-reactive CD8^+^ effector T cells capable of recognizing tumor-associated targets.

Our data extend these observations showing that OncoMimic-based immunotherapies can diversify convergent TCR patterns in adrenal cancers, particularly when combined with PD-1 checkpoint blockade. The diversification of shared TCR clusters across multiple patients further supports that EO2401 boosts reproducible, HLA class I-restricted immune responses. Multiple lines of evidence suggest that the EO2401-associated cluster signature reflects a genuine vaccine-driven imprint. Shared CDR3 motifs were detected both in vivo and after in vitro EO2401 stimulation (without nivolumab), with AC-specific enrichment and diversification through recruitment of additional clonotypes, dominated by TRBV6, TRBV29-1, and TRBV15. The observation that EO2401 broadens pre-existing cluster architectures by recruiting new clonotypes, rather than amplifying a fixed set of clones, is conceptually compatible with epitope spreading and bystander activation. In these mechanisms, an initial vaccine-directed T cell response progressively recruits additional clonotypes and antigen specificities, and such repertoire diversification is increasingly recognized not only as a hallmark of effective cancer vaccines and oncolytic immunotherapies, but also as a key feature of non-cancer vaccines contributing to anti-tumor immunity^36–39^. Notably, many of these clusters exhibited characteristics of public clonotypes - TCRs arising from germline recombination biases - that have been linked to effective antiviral and antitumor immunity^40,41^. This is also in line with studies showing that human tumors harbor abundant bystander T cells with viral specificities, including EBV-, CMV- and influenza-reactive T cells, and that melanoma-infiltrating non-tumor-reactive T cells are enriched for viral specificities with memory-like phenotypes^42–45^. Importantly, Rosato et al. and Çuburu et al. demonstrated that pre-existing antiviral memory T cells can be therapeutically repurposed or locally mobilized through viral epitope delivery to mediate anti-tumor activity, providing a mechanistic precedent for the emergence of viral-specific TCR clusters after EO2401 as a vaccine-associated recruitment of pathogen-imprinted immunity rather than nonspecific repertoire noise^44,45^. These conserved TCR features offer promising avenues for biomarker development and patient stratification in future immunotherapy trials.

Nevertheless, the extent of EO2401-induced repertoire convergence in this study was moderate despite the shared HLA-A*02 background among participants. A plausible explanation lies in the markedly restricted T cell architecture of the patient population characterized by significantly reduced clonal richness and moderately diminished diversity. This finding aligns with prior studies that characterize AC as an immune-sparse malignancy with low tumor-infiltrating lymphocyte density and limited immunogenicity, often exacerbated by glucocorticoid overproduction, which can systemically suppress T cell activity^46,47^. Additional factors may contribute to the limited convergence of EO2401-induced responses. Even within an HLA-matched cohort, interindividual variability in TCR repertoires, prior microbial exposure, and T cell precursor frequencies can shape the magnitude and quality of responses to immunotherapies. Differences in antigen processing and presentation efficiency, either due to tumor-intrinsic alterations in the antigen presentation machinery or heterogeneity in the expression of the targeted TAAs, may further impact T cell activation. Moreover, the design of EO2401 as a multi-epitope immunotherapy introduces a potential for immunodominance hierarchies, wherein individual patients preferentially respond to one mimetic peptide over others. Such patient-specific immunodominance can lead to divergent clonal expansions and reduce the frequency of clonotype overlap at the cohort level. Despite these challenges and the overall immunologically constrained patient population marked by its restricted repertoire, the observation of convergent TCR clusters, some shared across up to five out of 11 individuals, highlights the potential of OncoMimic peptides to drive public immune responses. Given the association between TIL density and improved survival in AC^47^, strategies that can enhance T cell infiltration and activation may have clinical potential.

Although the reported findings are promising, our study has limitations. The cohort size is small, immunosuppressed ACs have lower T cell numbers, which affects sequencing depth, and clinical correlations with response durability or tumor regression remain to be fully established. Additionally, while TCR sequencing revealed immunogenetic footprints of EO2401 administration, functional characterization of these expanded clonotypes through antigen-dependent cytotoxicity assays, single-cell TCR cloning, or tumor reactivity tests using patient-derived material is needed to confirm their antitumor potential as described for mouse models and healthy T cells^16^. In addition, some of the AC-associated repertoire features may also be influenced by anti-PD1-induced repertoire dynamics. Disentangling the individual contributions of the AC tumor environment, EO2401 vaccination, and checkpoint blockade will require future studies with dedicated control arms.

In conclusion, this study provides evidence that an OncoMimic-based strategy can elicit shared T cell responses in patients with adrenal cancer, despite the typically suppressed or low-diversity baseline TCR repertoire in these patients. These findings support the further clinical development of OncoMimic-based immunotherapies in combination with immune checkpoint inhibitors, potentially expanding the arsenal of immunotherapeutic strategies for rare and aggressive cancers.

## Supplementary information

Transparent Peer Review file

Supplementary_Information

Description of Additional Supplementary files

Supplementary_Data_1

Supplementary_Data_2

Supplementary_Data_3

Supplementary_Data_4

## Data Availability

Immune repertoire sequencing data is deposited in the European Nucleotide Archive (ENA, ID: PRJEB113632). Primary data for Fig. 1 is provided as Supplementary Data 1. GLIPH2 clusters are provided in Supplementary Tables 1, 2. GLIPH2 motifs matching CDR3 amino acid sequences from VDJdb, IEDB and neoTCR databases are provided as Supplementary Data 2–4. All other data used and/or analyzed in the current study are available from the corresponding author on reasonable request.
