# Supplementary material for: OncoMimic peptide-based immunotherapy EO2401 induces shared T cell clusters in patients with adrenal cancer
Source: Commun Med (Lond). 2026 Jul 6;6:569. doi: 10.1038/s43856-026-01755-8 (PMC13720076; doi:10.1038/s43856-026-01755-8)
Supplement: Supplementary file 1 [file 43856_2026_1755_MOESM1_ESM.pdf]

nature portfolio

Peer Review File

OncoMimic peptide-based immunotherapy EO2401 induces shared T cell clusters in patients with adrenal cancer

Corresponding Author: Professor Mascha Binder

This file contains all reviewer reports in order by version, followed by all author rebuttals in order by version.

Version 0:

Reviewer comments:

Reviewer #1

(Remarks to the Author)
The manuscript by Schultheiß et al. investigates the immune impact of EO2401, a microbiome-derived peptide immunotherapy, in patients with adrenal cancers. The authors performed longitudinal TCR sequencing together with in vitro stimulation and clustering analyses to characterize treatment-associated T cell responses. Overall, this is an interesting and relevant study. The idea of leveraging these microbial peptide mimics to mobilize pre-existing T cell immunity is appealing, particularly in adrenal cancers where immunotherapy responses are generally limited. The dataset, comprising longitudinal TCR repertoires from a rare cohort of adrenal cancer patients, provides a valuable resource for exploring repertoire dynamics following EO2401 treatment. However, I have several concerns regarding the interpretation/n of these data, which are largely computational and lack direct functional validation, particularly with respect to claims about antigen specificity and tumor relevance. The specific comments are listed as follows.
1. Antigen specificity is not clearly established
A central conclusion of the study is that EO2401 induces tumor-relevant, antigen-specific T cell responses. However, this is not directly demonstrated by the current data. The identification of shared clonotypes and GLIPH2 clusters is interesting, but these approaches are indirect. Given the known bias toward public TCRs with high generation probability, it is difficult to exclude that part of the observed expansion reflects intrinsic repertoire features rather than EO2401-driven selection. This concern is reinforced by the lack of consistent expansion of specific clonotypes across patients. Overall, the data appear more consistent with repertoire modulation than with clear antigen-specific responses.
2. Functional relevance of expanded T cells remains unclear
Related to the above point, the study does not address whether the expanded T cells are functionally relevant. It is not shown whether these T cells respond to EO2401 peptides, produce effector cytokines, or recognize tumor targets. Without such data, it is difficult to evaluate the biological significance of the observed repertoire changes.
3. Baseline comparisons require further clarification (Fig. 1)
The comparison between adrenal cancer, HNSCC, and healthy controls is informative, but it is not clear how well these cohorts are matched. TCR repertoire metrics can be influenced by sequencing depth and other technical variables. More detail is needed to assess whether the observed differences reflect biological variation. In addition, since all analyses are based on peripheral blood, the relevance to tumor-specific immune responses should be discussed more carefully.
4. Interpretation of shared clonotypes and clustering (Fig. 3)
The analysis of shared clonotypes and GLIPH2 clusters is central to the manuscript. While some TCR clusters are shared across patients, most clonotype sharing appears to be intra-patient, and consistent expansion across the cohort is limited. The authors should more clearly acknowledge the moderate degree of convergence when interpreting these results as evidence of a “public” T cell response
5. In vitro stimulation experiments are difficult to interpret (Fig. 4)
The in vitro stimulation experiments are intended to support EO2401-specific expansion. However, the observed changes in clonality and diversity could also be explained by general T cell expansion during stimulation. In addition, it is not clear whether appropriate controls (non-relevant peptide, health PBMC) were included. Without such controls, it is difficult to distinguish antigen-specific effects from non-specific proliferation. The overlap between in vitro and in vivo clusters is interesting, but still indirect and should be interpreted with caution.
6. Introduction could better define the knowledge gap
The Introduction provides a general overview of microbiome-associated immune modulation and the rationale for EO2401. However, the specific knowledge gap addressed by this study is not clearly defined. For example, it remains unclear how pre-existing T cells in cancer patients respond to microbial mimic peptides, whether these responses are reproducible

across individuals, and to what extent EO2401 can overcome the immune-cold features of adrenal cancers. Explicitly stating what remains unknown and how the current study addresses these gaps would strengthen the rationale and help the reader understand the study's significance.
7. Discussion may overinterpret antigen specificity
In the Discussion, the authors interpret the observed TCR clustering and expansion as evidence of antigen-specific, tumor-relevant immune responses. Given that the current data are largely indirect and based on repertoire analysis, this interpretation seems somewhat strong. A more cautious discussion acknowledging alternative explanations (e.g., expansion of public TCRs or non-specific activation) would improve the balance of the manuscript.
8. Additional limitations should be discussed more explicitly
While the current study provides interesting insights into EO2401-associated TCR repertoire changes, direct functional validation of the identified T cell clusters is lacking. The authors could consider discussing in the limitations section potential approaches such as single-TCR cloning, antigen-dependent cytotoxicity assays (including antigen knockout or pathway mapping), and assessing tumor relevance through tumor-infiltrating lymphocyte analyses or comparison of blood versus tumor TCR repertoires. Including these considerations would clarify the mechanistic and translational scope of the findings.
Minor points:
1. The use of parametric statistical tests (e.g., t-test) may not always be appropriate given the small sample size.
2. The definitions of “clonality” and “shared clonotypes” should be clarified.

Reviewer #2

(Remarks to the Author)
Yang et al. investigated peripheral TCRβ repertoire changes in patients with adrenal cancer treated with the EO2401 OncoMimic peptide-based immunotherapy in combination with nivolumab. This is a well-organized and interesting study. In particular, the detailed immune repertoire analysis before and after vaccination in such a rare tumor setting is valuable. The authors show that, although broad repertoire reshaping was limited, EO2401 treatment was associated with the expansion of shared T cell clusters with features of public clonotypes. The study is technically up to date and addresses an important immunological question in a rare disease. Overall, I think this manuscript is suitable for publication after minor revision.

Comments
The main strength of this manuscript is the careful TCR repertoire analysis in a rare tumor cohort. The finding that EO2401 treatment is associated with expansion of shared T cell clusters, rather than clear enrichment of identical individual clonotypes, is interesting and should be appreciated as such. At the same time, I think the authors should state this point very clearly and consistently throughout the manuscript, because the data support convergence mainly at the cluster level inferred by GLIPH2/OLGA, rather than strong convergence at the single clonotype level. This clarification would help readers understand the main message more precisely.

The manuscript would benefit from a slightly more explicit discussion of the functional limitation of the current study. The repertoire data and the ex vivo peptide stimulation results are supportive, but the direct evidence that the expanded clonotypes or clusters are truly tumor-reactive remains limited. The authors already mention that functional characterization of expanded clonotypes is needed, and I agree with this point. If any additional functional data are available, it would strengthen the manuscript to include them. For example, did the authors perform any TCR-T–based functional validation, or any direct tumor-reactivity assay using patient-derived material? If such experiments were not performed, this should be stated more clearly as a limitation.

Because EO2401 was given in combination with nivolumab, I think the Discussion should be a little more balanced regarding attribution of the observed repertoire changes. The current data are consistent with an EO2401-associated immunogenetic imprint, especially with the overlap between in vivo and ex vivo expanded clusters, but it remains difficult to fully separate the contribution of EO2401 from the effect of concomitant PD-1 blockade. A short and clear statement on this point in the Discussion would improve the interpretation.

Version 1:

Reviewer comments:

Reviewer #1

(Remarks to the Author)
I appreciate the authors' efforts in reanalyzing the data and addressing the comments raised in my initial review. I am satisfied that my concerns have been adequately addressed and have no further comments. I support acceptance of the manuscript in its current form.

Reviewer #2

(Remarks to the Author)
The authors have adequately addressed my comments. In particular, they have clarified that the observed convergence is mainly at the TCR cluster/motif level rather than at the individual clonotype level, and they have more explicitly acknowledged the lack of direct functional validation of the expanded clonotypes or clusters. The revised Discussion also provides a more balanced interpretation regarding the possible contribution of concomitant PD-1 blockade. I have no further

major concerns and support acceptance of the manuscript.

Open Access This Peer Review File is licensed under a Creative Commons Attribution 4.0 International License, which permits use, sharing, adaptation, distribution and reproduction in any medium or format, as long as you give appropriate credit to the original author(s) and the source, provide a link to the Creative Commons license, and indicate if changes were made.
In cases where reviewers are anonymous, credit should be given to 'Anonymous Referee' and the source.
The images or other third party material in this Peer Review File are included in the article's Creative Commons license, unless indicated otherwise in a credit line to the material. If material is not included in the article's Creative Commons license and your intended use is not permitted by statutory regulation or exceeds the permitted use, you will need to obtain permission directly from the copyright holder.
To view a copy of this license, visit https://creativecommons.org/licenses/by/4.0/

Reviewers' comments:

Reviewer #1 (Remarks to the Author):

The manuscript by Schultheiß et al. investigates the immune impact of EO2401, a microbiome-derived peptide immunotherapy, in patients with adrenal cancers. The authors performed longitudinal TCR sequencing together with in vitro stimulation and clustering analyses to characterize treatment-associated T cell responses. Overall, this is an interesting and relevant study. The idea of leveraging these microbial peptide mimics to mobilize pre-existing T cell immunity is appealing, particularly in adrenal cancers where immunotherapy responses are generally limited. The dataset, comprising longitudinal TCR repertoires from a rare cohort of adrenal cancer patients, provides a valuable resource for exploring repertoire dynamics following EO2401 treatment. However, I have several concerns regarding the interpretation/n of these data, which are largely computational and lack direct functional validation, particularly with respect to claims about antigen specificity and tumor relevance. The specific comments are listed as follows.

We thank the reviewer for the positive overall assessment and for the careful and constructive critique. We have re-analyzed our data accordingly, integrated published functional data on EO2401/OncoMimic peptides, integrated additional new analyses to characterize the observed TCR clusters, and softened our interpretation where indicated. Detailed responses to the single points are provided in the following.

1. Antigen specificity is not clearly established. A central conclusion of the study is that EO2401 induces tumor-relevant, antigen-specific T cell responses. However, this is not directly demonstrated by the current data. The identification of shared clonotypes and GLIPH2 clusters is interesting, but these approaches are indirect. Given the known bias toward public TCRs with high generation probability, it is difficult to exclude that part of the observed expansion reflects intrinsic repertoire features rather than EO2401-driven selection. This concern is reinforced by the lack of consistent expansion of specific clonotypes across patients. Overall, the data appear more consistent with repertoire modulation than with clear antigen-specific responses.

We thank the reviewer for raising this important point and agree that immunosequencing alone cannot formally prove cognate antigen specificity at the single-clonotype level. We also want to stress that the aim of our study is the description of the immunogenetic TCR-repertoire footprints of adrenal cancer and of EO2401 vaccination.
Direct functional CD8 read-outs (ELISpot, MHC-multimer staining, intracellular cytokine staining, cytotoxicity) for EO2401 have been generated reported separately for SPENCER and for the sister trial in glioblastoma (ROSALIE) and were the basis of the original platform paper by Talpin et al. (2025, J Immunother Cancer, ref. 16). To bring these data more clearly to the reader we now do the following:

- **We toned down phrasing for antigen specificity and now describe the TCR-level findings more consistently as a "EO2401-associated immunogenetic imprint at the cluster level" throughout the text.**
- **We now explicitly summarize the available functional evidence for EO2401 in a dedicated paragraph of the discussion: (i) in the platform paper (Talpin et al. 2025), each EO2401 mimic peptide was shown to elicit cross-reactive, IFN-γ-producing and cytotoxic CD8 T cells against the corresponding tumor-associated antigen (BIRC5,**

FOXM1, IL13RA2) in vitro from healthy donors and in HLA-A2 transgenic mice; (ii) in the ROSALIE glioblastoma trial, EO2401-specific CD8 T cells were detected by ELISpot in 100 % of evaluable patients and by tetramer staining ex vivo in 19/20 patients with confirmed cross-reactivity against the cognate TAAs in 20/21 patients; (iii) in SPENCER, the magnitude of the CD8 response against EO2401 peptides correlated with depth of radiological tumor shrinkage (Enterome/SPENCER ESMO 2023). These data establish that EO2401 induces functional, cross-reactive CD8 effector cells. Our repertoire data add the in-hominis molecular signature of these responses.

- To directly address the "public-TCR bias" concern we performed several re-analyses of the existing data, now presented in new Figure 5: (i) a comparison of OLGA-derived generation probabilities of vaccination-expanded versus baseline-background clonotypes, which were indistinguishable (Mann-Whitney $p = 0.85$, Cliff's $\delta = 0.007$; Figure 5B), excluding a bias toward intrinsically high-probability public clones; (ii) a cumulative motif-frequency analysis against 200 random length-matched control k-mers, demonstrating that adrenal-cancer selectivity is confined to the convergently recovered shared clusters (Figure 5C); and (iii) a convergence analysis showing that 63 % of the in vitro EO2401-stimulation clusters are independently recovered in vivo (32/51; hypergeometric $p < 1\times10^{-30}$; Figure 5A).,

2. Functional relevance of expanded T cells remains unclear. Related to the above point, the study does not address whether the expanded T cells are functionally relevant. It is not shown whether these T cells respond to EO2401 peptides, produce effector cytokines, or recognize tumor targets. Without such data, it is difficult to evaluate the biological significance of the observed repertoire changes.

We agree with the reviewer in general and have rebalanced the manuscript. Please also refer to the answer to point 1 above. Functional read-outs for EO2401 (IFN-γ ELISpot, tetramer staining, cytotoxicity against TAA-pulsed and HLA-A2+ tumor cell lines) have been generated as part of the EO2401 development program and the parental OncoMimic platform (Talpin et al. 2025, ref. 16) and have been previously communicated for the SPENCER and ROSALIE trials. The novelty of the present manuscript is the immunogenetic correlate: the same OncoMimic platform leaves a reproducible, partly public TCR-repertoire footprint in patients in vivo, and that this footprint is recapitulated by in-vitro stimulation with the EO2401 peptide pool. We now state this scope more clearly in introduction and discussion, and reference the available functional data.
In addition, several recent cancer-vaccine and oncolytic-virus studies have shown that vaccination-induced expansion of an initial set of antigen-specific clonotypes can drive bystander activation (including TCRs with viral specificities) and broaden anti-tumor immunity through epitope spreading. This mechanism is compatible with the moderate clonotype-level but reproducible cluster-level convergence we observe, namely the recruitment of additional CDR3 clonotypes into pre-existing cluster architectures (Figure 5D). We now address these points in more detail in the Discussion

3. Baseline comparisons require further clarification (Fig. 1). The comparison between adrenal cancer, HNSCC, and healthy controls is informative, but it is not clear how well these cohorts are matched. TCR repertoire metrics can be influenced by sequencing depth and other technical variables. More detail is needed to assess whether the observed differences reflect

biological variation. In addition, since all analyses are based on peripheral blood, the relevance to tumor-specific immune responses should be discussed more carefully.

We thank the reviewer for raising this important point and have expanded the methodological details in the revised manuscript. All cohorts were processed using the identical multiplex TRB protocol (250 ng gDNA input) within the same established processing and analysis pipeline. Productive reads were proportionally normalized to 50,000 reads per sample prior to downstream analyses without clonotype filtering. To improve transparency, we now provide per-sample read counts (Supplementary Figure 1A) and a new Supplementary Table 1 including raw read counts, repertoire metrics, and available demographic variables (age and sex), which are also summarized in the revised Table 1.

The SPENCER and healthy donor cohorts are reasonably age-matched, whereas the HNSCC cohort is older and male-dominated. Although sex information was unavailable for the SPENCER cohort, we selected the healthy donor cohort based on the known female predominance and peak incidence age of adrenal cancer. These cohort characteristics are now discussed more explicitly.

We also note that a subset of the adrenal cancer cohort showed lower read counts, which is biologically plausible given the immunosuppressive and frequently cortisol-secreting nature of these tumors. To address the potential impact of sequencing depth on repertoire metrics, we performed a rarefaction analyses (new Supplementary Figure 1B-C). These showed that Shannon diversity followed similar trajectories across cohorts when assessed as clonotypes accumulated, and that richness increased with sequencing depth as expected for bulk TCR sequencing, without evidence that cohort-level differences were primarily driven by undersampling. Together, these analyses support the robustness of the normalized repertoire comparisons while acknowledging incomplete capture of very rare clonotypes.

Finally, we agree that peripheral blood does not directly capture tumor-infiltrating immune responses. We have therefore clarified in the discussion that our study specifically assesses systemic immune repertoire alterations associated with disease rather than tumor-localized T cell responses.

4. Interpretation of shared clonotypes and clustering (Fig. 3). The analysis of shared clonotypes and GLIPH2 clusters is central to the manuscript. While some TCR clusters are shared across patients, most clonotype sharing appears to be intra-patient, and consistent expansion across the cohort is limited. The authors should more clearly acknowledge the moderate degree of convergence when interpreting these results as evidence of a “public” T cell response.

We fully agree and we have rephrased abstract, results and discussion to clearly distinguish convergence at the clonotype level from convergence at the cluster/motif level.

5. In vitro stimulation experiments are difficult to interpret (Fig. 4). The in vitro stimulation experiments are intended to support EO2401-specific expansion. However, the observed

changes in clonality and diversity could also be explained by general T cell expansion during stimulation. In addition, it is not clear whether appropriate controls (non-relevant peptide, health PBMC) were included. Without such controls, it is difficult to distinguish antigen-specific effects from non-specific proliferation. The overlap between in vitro and in vivo clusters is interesting, but still indirect and should be interpreted with caution.

We thank the reviewer for raising this point. We acknowledge that the assay was not designed as a head-to-head antigen specificity assay but as a stress test of the in vivo repertoire findings. While we cannot completely exclude enrichment patterns mediated by selective proliferation or dying of distinct T cells in vitro, the comparative analysis of the post-vaccination and in vitro stimulation patterns using the identical peptide pool provides a plausible approximation. Notably, the fact that 63 % of the GLIPH2 clusters identified after ex vivo EO2401 peptide stimulation are also independently recovered in the in vivo post-vaccination repertoires (32/51; hypergeometric $p < 1\times10^{-30}$; new Figure 5A) is a strong argument that the in vivo signature is peptide-driven and not non-specific.

6. Introduction could better define the knowledge gap. The Introduction provides a general overview of microbiome-associated immune modulation and the rationale for EO2401. However, the specific knowledge gap addressed by this study is not clearly defined. For example, it remains unclear how pre-existing T cells in cancer patients respond to microbial mimic peptides, whether these responses are reproducible across individuals, and to what extent EO2401 can overcome the immune-cold features of adrenal cancers. Explicitly stating what remains unknown and how the current study addresses these gaps would strengthen the rationale and help the reader understand the study's significance.

We appreciate this suggestion. We restructured the introduction accordingly and now explicitly mention knowledge gaps in the field.

7. Discussion may overinterpret antigen specificity. In the Discussion, the authors interpret the observed TCR clustering and expansion as evidence of antigen-specific, tumor-relevant immune responses. Given that the current data are largely indirect and based on repertoire analysis, this interpretation seems somewhat strong. A more cautious discussion acknowledging alternative explanations (e.g., expansion of public TCRs or non-specific activation) would improve the balance of the manuscript.

We agree that a careful wording is necessary for the interpretation of the presented data. We rephrased the discussion accordingly and want to especially highlight the following key points added:

- **The repertoire footprint we describe is consistent with but does not by itself prove antigen-specific cross-reactive CD8 responses against BIRC5/FOXM1/IL13RA2.**
- **Part of the observed convergence may reflect intrinsic HLA-A2 public-TCR biases; however, the new analyses in Figure 5 (The indistinguishable generation-probability distributions of expanded versus background clonotypes (Figure 5B) and the absence of enrichment of random length-matched control motifs (Figure 5C)) argue against this being the sole explanation.**

- **In addition, we now describe a recruitment of new low-frequency CDR3 clones to distinct TCR clusters after EO2401 vaccination (Figure 5D), in line with a broadening of the immune response.**
- **BecauseEO2401 was administered concomitantly with nivolumab, a contribution of PD-1 blockade to global repertoire reshaping cannot be excluded (see also Reviewer #2, point 3); however, the in vitro recapitulation of 63 % of the in vivo clusters by the EO2401 peptide pool alone, in the absence of nivolumab (Figure 5A), argues for a substantial EO2401-attributable component of the signature.**
- **We now also include an advanced data base profiling (VDJdb, IEDB, neoTCR) for CDR3 amino acid sequences matching the detected cluster motifs (Figure 5E-F; Supplemental Tables 4-6). While we did not observe a clear convergence on TCRs with known TAA/neoantigen specificity, we observed many hits in line with broader pathogen-imprinted/public TCR architectures. Bystander activation and epitope spreading are well-described downstream effects of effective peptide vaccines and may further blur the line between strictly antigen-specific and vaccine-associated repertoire shifts nevertheless are an important mechanism of anti-tumor efficacy.**

8. Additional limitations should be discussed more explicitly. While the current study provides interesting insights into EO2401-associated TCR repertoire changes, direct functional validation of the identified T cell clusters is lacking. The authors could consider discussing in the limitations section potential approaches such as single-TCR cloning, antigen-dependent cytotoxicity assays (including antigen knockout or pathway mapping), and assessing tumor relevance through tumor-infiltrating lymphocyte analyses or comparison of blood versus tumor TCR repertoires. Including these considerations would clarify the mechanistic and translational scope of the findings.

We thank the reviewer for this suggestion and extended the limitations section accordingly including future directions.

Minor points:
1. The use of parametric statistical tests (e.g., t-test) may not always be appropriate given the small sample size.

As suggested, we replaced the t-test in Figure 2 with the Wilcoxon signed-rank for paired BL/FU comparisons. Figure legends have been updated accordingly. The pattern and significance of the results are unchanged. Notably, a one-way ANOVA with Tukey's post-hoc test (not t-test) was used in Figure 1A. We apologize for this error.

2. The definitions of "clonality" and "shared clonotypes" should be clarified.

We tried to provide more precise definitions of these points: As stated in the methods section, clonality is defined as 1-Pielou's evenness index (Shannon entropy divided by the natural logarithm of the number of unique productive CDR3 clonotypes per sample) and ranges from 0 (perfectly even repertoire) to 1 (single dominating clone). A clonotype is defined as an unique productive CDR3 nucleotide sequence. A shared clonotype is defined as an identical productive CDR3 amino acid sequence detected in samples from ≥ 2

individuals (inter-patient sharing) or ≥ 2 time points of the same individual (intra-patient sharing).

Reviewer #2 (Remarks to the Author):
Yang et al. investigated peripheral TCRβ repertoire changes in patients with adrenal cancer treated with the EO2401 OncoMimic peptide-based immunotherapy in combination with nivolumab. This is a well-organized and interesting study. In particular, the detailed immune repertoire analysis before and after vaccination in such a rare tumor setting is valuable. The authors show that, although broad repertoire reshaping was limited, EO2401 treatment was associated with the expansion of shared T cell clusters with features of public clonotypes. The study is technically up to date and addresses an important immunological question in a rare disease. Overall, I think this manuscript is suitable for publication after minor revision.

We thank the reviewer for the favorable assessment of our work. We address all comments in the following.

Comments
The main strength of this manuscript is the careful TCR repertoire analysis in a rare tumor cohort. The finding that EO2401 treatment is associated with expansion of shared T cell clusters, rather than clear enrichment of identical individual clonotypes, is interesting and should be appreciated as such. At the same time, I think the authors should state this point very clearly and consistently throughout the manuscript, because the data support convergence mainly at the cluster level inferred by GLIPH2/OLGA, rather than strong convergence at the single clonotype level. This clarification would help readers understand the main message more precisely.

We fully agree with the reviewer. As also detailed above in response to Reviewer #1 (major points 4 and 7), we have rephrased the abstract, results and discussion to make this distinction explicit and consistent throughout the manuscript.

The manuscript would benefit from a slightly more explicit discussion of the functional limitation of the current study. The repertoire data and the ex vivo peptide stimulation results are supportive, but the direct evidence that the expanded clonotypes or clusters are truly tumor-reactive remains limited. The authors already mention that functional characterization of expanded clonotypes is needed, and I agree with this point. If any additional functional data are available, it would strengthen the manuscript to include them. For example, did the authors perform any TCR-T–based functional validation, or any direct tumor-reactivity assay using patient-derived material? If such experiments were not performed, this should be stated more clearly as a limitation.

These are important points and which are now more clearly acknowledged in the revised manuscript. Unfortunately, functional assays were not feasible in the trial context given the limited material available. We now discuss this more clearly in the limitation section. As also outlined above in response to Reviewer #1 we now also more clearly address EO2463-releated functional findings from other studies, in particular the functional CD8 read-outs for EO2401 (IFN-γ ELISpot, MHC-multimer staining, cytotoxicity against TAA-pulsed targets) that have been published in the parental OncoMimic platform paper (Talpin et al., J

Immunother Cancer 2025, ref. 16) and reported for the ROSALIE glioblastoma trial and SPENCER itself (ESMO 2023: correlation between CD8 response magnitude and degree of radiological tumor shrinkage).

Because EO2401 was given in combination with nivolumab, I think the Discussion should be a little more balanced regarding attribution of the observed repertoire changes. The current data are consistent with an EO2401-associated immunogenetic imprint, especially with the overlap between in vivo and ex vivo expanded clusters, but it remains difficult to fully separate the contribution of EO2401 from the effect of concomitant PD-1 blockade. A short and clear statement on this point in the Discussion would improve the interpretation.

We thank the reviewer for raising this important point. The argument that allows us to attribute at least a substantial component of the observed signature to EO2401 rather than to nivolumab alone is the in-vitro experiment of Figure 4: peripheral blood mononuclear cells from the same patients, stimulated with the EO2401 peptide pool in the complete absence of nivolumab, recover 63 % of the in vivo GLIPH2 clusters (32/51; hypergeometric $p < 1\times10^{-30}$; new Figure 5A). This indicates that a substantial component of the in vivo signature is EO2401-attributable rather than a generic PD-1-blockade effect. Conversely, we fully agree with the reviewer that quantitative global repertoire features (richness, diversity drift, V-gene usage trends) are likely influenced by nivolumab as well, in line with published anti-PD-1-induced repertoire dynamics, and that disentangling individual contributions will require dedicated control arms in future trials. We expanded the Discussion in the revised manuscript accordingly.
