# Supplementary material for: OncoMimic peptide-based immunotherapy EO2401 induces shared T cell clusters in patients with adrenal cancer
Source: Commun Med (Lond). 2026 Jul 6;6:569. doi: 10.1038/s43856-026-01755-8 (PMC13720076; doi:10.1038/s43856-026-01755-8)
Supplement: Supplementary file 2 [file 43856_2026_1755_MOESM2_ESM.pdf]

**SUPPLEMENTARY INFORMATION**

# OncoMimic peptide-based immunotherapy EO2401 induces shared T cell clusters in patients with adrenal cancer

Christoph Schultheiß[1,2], Brenda Besemer[1,2], Edith Willscher[3], Lisa Paschold[3], Tifanny Mersceman[5], Alice Talpin[5], Clara Serger[1,4], Alfred Zippelius[1,4], Alfredo Berruti[6], Salvatore Grisanti[6], Catharina Willemien Menke-van der Houven van Oordt[7], Eric Baudin[8], Laura-Sophie Landwehr[9], Jaume Capdevila[10], Vivek Subbiah[11], Dan Granberg[12], Kirsten Gedske Daugaard[13], Alexandra Triebig[14], Thibault Gauduchon[15], Christine Do Cao[16], Marie-Eve Garcia[17], Joao Magalhaes[5], Laurent Chêne[5], Mascha Binder[1,2,18]

[1] Division of Medical Oncology, University Hospital Basel; Basel, Switzerland.
[2] Laboratory of Translational Immuno-Oncology, Department of Biomedicine, University and University Hospital Basel; Basel, Switzerland.
[3] Department of Internal Medicine IV, Oncology/Hematology, Martin-Luther-University Halle-Wittenberg, Halle (Saale), Germany.
[4] Laboratory of Cancer Immunology, Department of Biomedicine, University Hospital and University of Basel, Basel, Switzerland.
[5] Enterome SA, Paris (France).
[6] Università degli Studi di Brescia, Azienda Ospedaliera Spedali Civili, Brescia, Italy.
[7] Department of Medical Oncology, Amsterdam UMC, Cancer Centre Amsterdam, The Netherlands.
[8] Endocrine Oncology, Gustave Roussy, Villejuif, France.
[9] Internal Medicine, University Hospital Würzburg, Würzburg, Germany.
[10] Vall d'Hebron University Hospital and Vall d'Hebron Institute of Oncology (VHIO), Barcelona, Spain.
[11] Stanford Cancer Institute, Stanford University School of Medicine, Stanford, CA, USA.
[12] Department of Molecular Medicine and Surgery, Karolinska Institute, Stockholm, Karolinska University Hospital Solna, Stockholm, Sweden.
[13] Medical Oncology, Rigshospitalet/University Hospital, Copenhagen, Denmark.
[14] Medicine IV Endocrine Oncology, Ludwig Maximilians University of Munich, München, Germany.
[15] Medical Oncology, Centre Léon Bérard, Lyon, France.
[16] University Hospital Lille - Hôpital Claude Huriez, Lille, France.
[17] Hospital Nord, Marseille, France.
[18] Collaborative Research Institute Intelligent Oncology (CRIION), Freiburg, Germany.

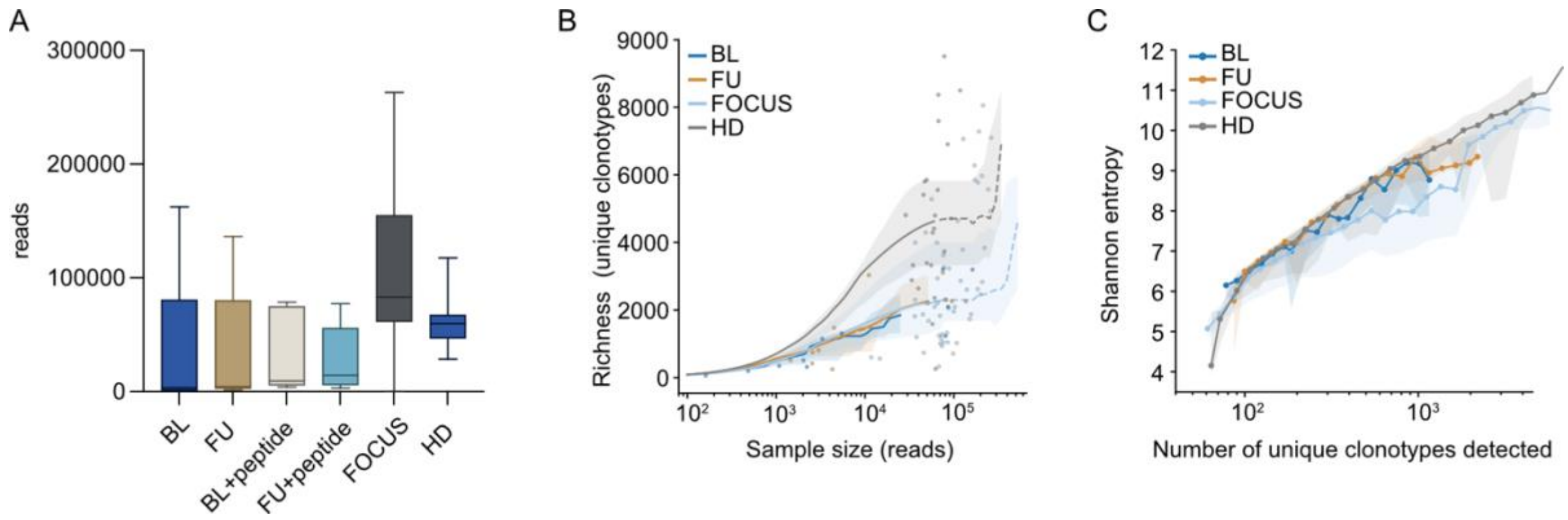


**Supplementary Figure 1. TCR sequencing reads and rarefaction analyses. (A)** Median read counts per cohort, boxes display interquartile range (IQR), whiskers indicate min to max values. **(B)** Rarefaction analysis of sequencing reads and number of unique clonotypes. Curves represent the per-cohort median, with shaded areas indicating the interquartile range (IQR). Solid lines show interpolation up to the observed sequencing depth, while dashed lines show extrapolation beyond the observed depth, capped at fivefold the observed read depth. Open circles indicate the observed endpoint for each individual sample, defined by its read count and observed clonotype richness. **(C)** Rarefaction analysis of Shannon entropy (diversity) and the number of unique clonotypes. Lines represent the per-cohort median from Monte Carlo sampling, with shaded areas indicating the IQR. Patient cohorts largely overlap, while healthy donors show slightly higher Shannon entropy at matched clonotype counts, suggesting a more even repertoire independent of clonotype richness.

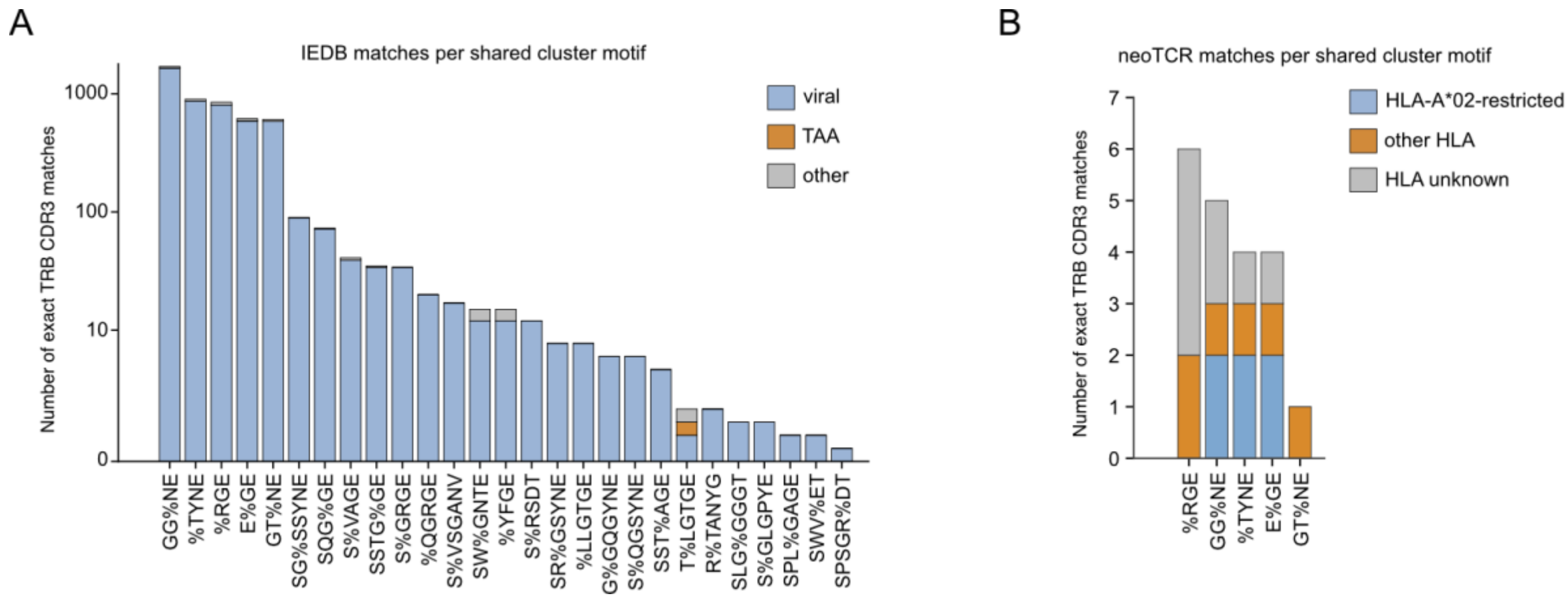


**Supplementary Figure 2. Cross-validation of VDJdb CDR3 matches with shared EO2401 cluster motif. (A)** IEDB cross-validation. The 32 cluster motifs shared between the in vivo and in vitro GLIPH2 catalogues (Figure 5A) were cross-referenced against the human TRBβ receptor content of the Immune Epitope Database (IEDB; receptor export tcr_full_v3, 187,488 human TRBβ receptors). Bars represent the number of matching IEDB CDR3β sequences per shared cluster motif, stratified by antigen category into viral, TAA (curated set, as in Figure 5F) and other (log-scaled y-axis). **(B)** NeoTCR cross-validation. The 32 cluster motifs shared between the in vivo and in vitro GLIPH2 catalogues (Figure 5A) were cross-referenced against NeoTCR, a curated database of experimentally-supported functional neoantigen-specific TCRs (988 entries across 17 cancer types; Zhou et al., Genomics Proteomics Bioinformatics 2025. Matching was performed for the 954 full-length CDR3β sequences, with the same rule as for VDJdb and IEDB. Bars represent the number of matching NeoTCR CDR3β sequences per shared cluster motif, stratified by the HLA restriction of the cognate neoepitope into HLA-A*02-restricted, other (defined non-A*02) HLA, and HLA not reported.

**Supplementary Table 1: T cell consensus sequences in adrenal cancer patients after vaccination with EO2401 compared to healthy individuals.***

| log2 pGen | Samples with cluster | Cluster presence per group (healthy. vacc. cancer pts) | Cluster enrichment in vacc. pts | Consensus |
|---|---|---|---|---|
| -29.4270388 | 2 | both | 23.18193196 | DRG%GEK |
| -24.3066799 | 2 | both | 16.84500339 | RE%GANV |
| -25.6907365 | 2 | vacc. patients only | 13.83792594 | %RGE |
| -25.6907365 | 2 | vacc. patients only | 13.83792594 | E%GE |
| -21.9974661 | 4 | both | 13.57107 | %GVNE |
| -26.6813175 | 2 | vacc. patients only | 12.82653891 | ST%AASTDT |
| -22.0325728 | 5 | both | 8.978654551 | S%RSDT |
| -21.8894057 | 3 | both | 8.976505423 | SSR%DT |
| -27.2198959 | 2 | both | 8.695629279 | SIDR%HNE |
| -24.3807441 | 4 | both | 6.774038248 | GG%NE |
| -28.064453 | 2 | both | 6.19936179 | SK%GQKET |
| -32.0148018 | 2 | both | 6.05683032 | SEMAG%EET |
| -28.4420085 | 2 | both | 5.07380659 | %APGQPYNE |
| -26.2956895 | 3 | both | 4.900202259 | SPW%GTGE |
| -27.5060054 | 2 | vacc. patients only | 3.825688507 | T%LGTGE |
| -22.1826768 | 2 | both | 3.747266444 | SS%GAGE |
| -25.4915519 | 3 | both | 3.735626488 | SSTG%GE |
| -20.7031093 | 3 | both | 3.689937315 | SQGR%E |
| -23.9745641 | 2 | both | 3.675272197 | SQ%RGE |
| -21.9407386 | 3 | both | 3.581807027 | SW%GNTE |
| -29.0369439 | 2 | vacc. patients only | 3.53059566 | %ASGGNNE |
| -22.6624309 | 2 | post | 3.450632965 | %LLGTGE |
| -27.2233877 | 2 | post | 3.202115159 | RP%GTGSYE |
| -27.2233877 | 4 | post | 3.202115159 | RPTG%GSYE |
| -27.2233877 | 3 | post | 3.202115159 | RPTGT%SYE |
| -28.5288613 | 2 | vacc. patients only | 3.162635858 | S%RSGGRET |
| -28.7244796 | 2 | both | 3.156421339 | SP%SGGRET |
| -25.4499884 | 4 | both | 3.036482121 | GT%NE |
| -22.5406528 | 3 | both | 3.034397528 | %TYNE |
| -23.0236052 | 2 | vacc. patients only | 3.020614516 | SPSGR%DT |
| -24.0361157 | 6 | both | 3.012927111 | G%YNE |
| -25.3790534 | 3 | both | 2.888917251 | R%TANYG |
| -30.0529246 | 2 | both | 2.885508186 | G%GQGYNE |
| -19.9465768 | 2 | both | 2.809452984 | SSRG%QP |
| -25.7896011 | 5 | both | 2.806286896 | SG%SSYNE |
| -24.6561482 | 4 | both | 2.651446911 | S%VAGE |
| -28.3319276 | 2 | vacc. patients only | 2.440955336 | SLG%GGGT |
| -22.7653473 | 4 | both | 2.362180288 | SY%SGSYE |
| -24.0520994 | 3 | both | 2.35903968 | SWV%ET |
| -25.7950729 | 2 | both | 2.278540165 | SPE%RGTDT |

| | | | | |
|---|---|---|---|---|
| -28.0434742 | 3 | both | 2.239473698 | SPL%GAGE |
| -23.4340176 | 4 | both | 2.152992332 | SPGLG%YE |
| -23.4340176 | 2 | both | 2.150703623 | S%GLGPYE |
| -27.2587203 | 2 | vacc. patients only | 1.936943036 | %YFGE |
| -26.8194167 | 4 | both | 1.911011243 | D%GTDT |
| -23.9460958 | 2 | vacc. patients only | 1.901193472 | %QGRGE |
| -27.8923234 | 5 | both | 1.89523074 | DRG%DT |
| -24.1372736 | 2 | vacc. patients only | 1.88995822 | SST%AGE |
| -22.983454 | 5 | both | 1.850434987 | S%GRGE |
| -22.6967935 | 5 | both | 1.846386269 | SQG%GE |
| -21.7642434 | 4 | both | 1.836685537 | S%QGSYNE |
| -22.772255 | 6 | both | 1.835299234 | SR%GSYNE |
| -23.4641169 | 3 | both | 1.816168894 | SRQG%YNE |
| -27.7033638 | 2 | both | 1.749514029 | SE%GDGANV |
| -24.4443689 | 2 | vacc. patients only | 1.744516981 | SPV%GANV |
| -23.3503649 | 2 | vacc. patients only | 1.742324512 | S%VSGANV |
| -21.2446638 | 3 | both | 1.723501548 | STT%E |
| -22.7371952 | 3 | both | 1.670253456 | S%YG |
| -27.4949599 | 2 | both | 1.661440411 | S%SGSAE |
| -25.6615394 | 2 | both | 1.660831738 | %NYG |
| -24.1644232 | 2 | both | 1.526392463 | %SRSYNE |

* Clusters correspond to those highlighted in Figure 2B. vacc. = vaccinated. pts = patients. pGEN = generation probability

**Supplementary Table 2: T cell consensus sequences in adrenal cancer patients at baseline and after EO2401 vaccination and ex-vivo expansion.***

| log2 pGen | Samples with cluster | Cluster presence per group (cancer/vacc. cancer pts) | Cluster enrichment in vacc. pts | Consensus |
|---|---|---|---|---|
| -25.6907365 | 2 | vacc. patients only | 13.83792594 | %RGE |
| -24.6944739 | 3 | both | 13.82728349 | E%GE |
| -26.4879046 | 2 | both | 13.52927316 | G%VNE |
| -34.5992272 | 2 | both | 13.16439331 | S%TGY |
| -23.2751984 | 2 | both | 8.986336446 | S%RSDT |
| -23.7216635 | 3 | both | 8.264839568 | SLAG%SP |
| -26.4407092 | 2 | both | 7.04043802 | SASGG%NE |
| -23.4962465 | 2 | vacc. patients only | 6.800999048 | GG%NE |
| -33.3769148 | 2 | both | 5.224747556 | EVG%GNE |
| -24.7476511 | 2 | both | 4.508836367 | SL%LSET |
| -25.1380212 | 2 | both | 4.327287975 | SFL%GGG |
| -25.2545465 | 2 | both | 3.858187641 | SD%AGNEK |
| -27.5060054 | 2 | vacc. patients only | 3.825688507 | T%LGTGE |
| -25.2707091 | 2 | both | 3.795899607 | SDR%GNEK |
| -23.1556372 | 3 | both | 3.755187066 | SSTG%GE |
| -21.9407386 | 3 | both | 3.572725838 | SW%GNTE |
| -29.0369439 | 2 | vacc. patients only | 3.53059566 | %ASGGNNE |
| -24.4973889 | 2 | both | 3.460148595 | %PVSGANV |
| -21.6650411 | 3 | both | 3.426133669 | %LLGTGE |
| -27.2233877 | 3 | vacc. patients only | 3.202115159 | RPTG%GSYE |
| -27.2233877 | 3 | vacc. patients only | 3.202115159 | RPTGT%SYE |
| -27.2233877 | 2 | vacc. patients only | 3.202115159 | RP%GTGSYE |
| -25.0762723 | 2 | both | 3.047387579 | %TYNE |
| -26.9578943 | 2 | both | 3.017018595 | G%GQGYNE |
| -25.4499884 | 2 | both | 2.988521614 | GT%NE |
| -22.0250522 | 3 | both | 2.949552675 | SPSGR%DT |
| -23.3421971 | 2 | both | 2.814704006 | SG%SSYNE |
| -26.2708855 | 2 | both | 2.769095179 | %GVSSYNE |
| -44.5990151 | 2 | both | 2.683388162 | %WGAGAG |
| -25.3790534 | 3 | both | 2.579313882 | R%TANYG |
| -24.9626381 | 2 | both | 2.485986832 | SPR%NQET |
| -28.3319276 | 2 | vacc. patients only | 2.440955336 | SLG%GGGT |
| -24.722403 | 2 | vacc. patients only | 2.382661262 | SWV%ET |
| -27.6807479 | 2 | both | 2.241660922 | SPL%GAGE |
| -23.575689 | 2 | both | 2.213871997 | SPLGGA%E |
| -23.4340176 | 2 | both | 2.150030794 | S%GLGPYE |
| -22.9992192 | 3 | both | 2.002727379 | S%VAGE |
| -27.2587203 | 2 | vacc. patients only | 1.936943036 | %YFGE |
| -23.9460958 | 2 | vacc. patients only | 1.901193472 | %QGRGE |
| -22.974576 | 3 | both | 1.859011617 | S%GRGE |

| | | | | |
|---|---|---|---|---|
| -21.6999421 | 4 | both | 1.843284571 | SR%GSYNE |
| -22.974576 | 5 | both | 1.822449664 | SQG%GE |
| -20.9282152 | 2 | both | 1.803072956 | SLD%P |
| -26.2784071 | 3 | both | 1.756677922 | SPSGS%E |
| -24.4443689 | 2 | vacc. patients only | 1.744516981 | SPV%GANV |
| -23.3503649 | 2 | vacc. patients only | 1.742324512 | S%VSGANV |
| -21.6999421 | 4 | both | 1.739622806 | S%QGSYNE |
| -24.1372736 | 4 | both | 1.718043299 | SST%AGE |
| -20.1361812 | 2 | both | 1.66011885 | SRDS%QP |
| -30.1369701 | 2 | both | 1.658776859 | R%TGTGNYG |
| -25.3512544 | 2 | both | 1.595223668 | RRLA%DT |

* Clusters correspond to those highlighted in Figure 2C. vacc. = vaccinated. pts = patients. pGEN = generation probability
