# Supplementary material for: OncoMimic peptide-based immunotherapy EO2401 induces shared T cell clusters in patients with adrenal cancer
Source: Commun Med (Lond). 2026 Jul 6;6:569. doi: 10.1038/s43856-026-01755-8 (PMC13720076; doi:10.1038/s43856-026-01755-8)
Supplement: Supplementary file 3 [file 43856_2026_1755_MOESM3_ESM.docx]

**Description of Additional Supplementary Files**

File name: Supplementary Data 1

Description: Primary data for Figure 1

File name: Supplementary data 2-4

Description: GLIPH2 motifs matching CDR3 amino acid sequences from VDJdb, IEDB and neoTCR databases
